# Regulation of Spike Timing-Dependent Plasticity of Olfactory Inputs in Mitral Cells in the Rat Olfactory Bulb

**DOI:** 10.1371/journal.pone.0035001

**Published:** 2012-04-19

**Authors:** Teng-Fei Ma, Xiao-Lei Zhao, Lei Cai, Nan Zhang, Si-Qiang Ren, Fang Ji, Tian Tian, Wei Lu

**Affiliations:** 1 Department of Neurobiology, Nanjing Medical University, Nanjing, Jiangsu Province, People's Republic of China; 2 Key Laboratory for Neurodegenerative Disease of Jiangsu Province, Nanjing Medical University, Nanjing, Jiangsu Province, People's Republic of China; 3 Laboratory of Reproductive Medicine, Nanjing Medical University, Nanjing, Jiangsu Province, People's Republic of China; 4 Key Laboratory for Human Functional Genomics of Jiangsu Province, Nanjing Medical University, Nanjing, Jiangsu Province, People's Republic of China; Neuroscience Campus Amsterdam, -VU University, The Netherlands

## Abstract

The recent history of activity input onto granule cells (GCs) in the main olfactory bulb can affect the strength of lateral inhibition, which functions to generate contrast enhancement. However, at the plasticity level, it is unknown whether and how the prior modification of lateral inhibition modulates the subsequent induction of long-lasting changes of the excitatory olfactory nerve (ON) inputs to mitral cells (MCs). Here we found that the repetitive stimulation of two distinct excitatory inputs to the GCs induced a persistent modification of lateral inhibition in MCs in opposing directions. This bidirectional modification of inhibitory inputs differentially regulated the subsequent synaptic plasticity of the excitatory ON inputs to the MCs, which was induced by the repetitive pairing of excitatory postsynaptic potentials (EPSPs) with postsynaptic bursts. The regulation of spike timing-dependent plasticity (STDP) was achieved by the regulation of the inter-spike-interval (ISI) of the postsynaptic bursts. This novel form of inhibition-dependent regulation of plasticity may contribute to the encoding or processing of olfactory information in the olfactory bulb.

## Introduction

At the first station of central odor processing, the main olfactory bulb (MOB), signal processing is regulated by synaptic interactions between glutamatergic and GABAergic inputs of the mitral cells and tufted cells (M/T cells), which are the major projection neurons. The M/T cells receive both excitatory glutamatergic inputs from the olfactory sensory neurons (OSNs) and inhibitory GABAergic inputs from the interneurons, which predominantly consist of periglomerular cells (PGCs) and GCs [1], [2]. The GABAergic inhibitory inputs from the interneurons deliver lateral or recurrent inhibition to the M/T cells to modulate incoming sensory inputs to an optimal level. Because the GCs outnumber the PGCs in the bulb and because only approximately 20% of the PGCs make connections with both presynaptic OSNs and postsynaptic M/T cells [3], [4], most of the lateral inhibition is mediated by the GCs [1], [5]. The lateral inhibition from the GCs is exerted via reciprocal dendrodendritic synaptic connections with the M/T cells and functions as a contrast enhancer to facilitate the discrimination of correlated ON inputs [6].

Long-term potentiation (LTP) and long-term depression (LTD) are two forms of synaptic plasticity that are considered as the cellular substrates for learning and memory [7], [8]. LTP or LTD in the OSN-MC synapses in the main olfactory bulb can be produced by a brief tetanic or low frequency ON stimulation [9], [10]. Recently, LTP of the field excitatory postsynaptic potentials (fEPSPs) in the glomerulus was induced by theta burst stimulation (TBS) of the ON [11]. Moreover, Hebbian spike timing-dependent plasticity (STDP), a form of plasticity that strictly requires a temporal correlation between the pre- and postsynaptic responses, was also induced in vivo in β–lobe neurons of the locust mushroom body through the electrical stimulation of Kenyon cells [12] and in the excitatory inputs to the GCs in the main rat olfactory bulb [13]. Despite these elegant investigations in different levels and species, there have been a lack of studies that directly examine STDP in the MCs. In the olfactory bulb, the lateral inhibition driven by the GCs tends to affect the dendritic depolarization induced by the excitatory inputs and either prevents or delays firing in the MCs or decreases the firing rate during stimulus presentation [14], [15]. This change in the firing rate begins at the end of the spiking period induced by odor stimulation in vivo [15], indicating that the recent activity history could affect lateral inhibition between the MCs. However, little is known about whether and how prior persistent modification of the inhibition modifies the predisposition for subsequent induction of long-lasting changes of the excitatory OSN-MC synapses. This form of synaptic plasticity regulation has not been fully investigated in the olfactory system. Because STDP is a form of synaptic plasticity whose induction is heavily dependent on the timing of the incoming spikes and the frequency of postsynaptic spikes when the spike bursts are induced by the pairing protocol [16], the presence of lateral inhibition onto the postsynaptic MCs may profoundly influence the induction of STDP and the total olfactory bulb output. Moreover, the timing of synaptic inhibition itself may also be regulated [17], [18]. Therefore, the lateral inhibition exerted by the GCs plays critical roles in odor information processing and in olfactory learning and memory [5], [6], [19], [20], [21], [22], [23]. The mechanism by which the frequency of the intra-burst spikes is controlled by lateral inhibition is of great importance in understanding how sensory information is encoded and processed.

GCs receive two types of excitatory inputs on their proximal and distal dendrites [13], [24]. The reciprocal dendrodendritic synapses with the MCs are the primary source of distal excitatory inputs, which mediate local dendrodendritic inhibition (DDI) [25]. Cortical feedback input is one of the major sources of proximal input and mediates the global top-down modulation of DDI in the olfactory bulb [24]. These two anatomically distinct excitatory inputs display persistent modification with opposing directions when subjected to the same stimulating protocol [13], which suggests that they may differentially exert their influence on the subsequent induction of synaptic plasticity in MC excitatory synapses and thus have different implications in regulating the total olfactory bulb output. Because the extent of granule cell (GC) function in the local versus global output modes can have an important impact on the computational role GC performs in the olfactory bulb circuit [1], the modification of the excitatory ON input by prior plasticity of the distinct excitatory inputs to GCs will profoundly influence the encoding and processing of odor information in the bulb.

In this study, we found that TBS could elicit differential plasticity of two different excitatory inputs to the GCs. Interestingly, the same protocol also induced a persistent modification in the lateral inhibition to the MCs with opposing directions. This bidirectional modification of the inhibitory inputs differentially regulated the predisposition of the subsequent induction of STDP of excitatory ON inputs to the MCs. Further evidence demonstrated that this regulation was achieved by the regulation of the spike frequency within the bursts employed by the pairing protocol for STDP induction. Thus, our results revealed one of the mechanisms by which the frequency of the intra-burst spikes is controlled in the sensory system. Because fine temporal burst structure is proposed to convey stimulus-related information to postsynaptic cells, this novel form of inhibition-dependent regulation of plasticity may contribute to the encoding or processing of olfactory information in the olfactory bulb.

## Materials and Methods

### Ethics Statement

The protocols for the animal care and use were approved by the Experimental Animal Ethics Committee at the Nanjing Medical University (permit number 20100582).

### Olfactory bulb brain slice preparation

Acute olfactory bulb slices were prepared from P14–21 Sprague Dawley rats. The rats were deeply anesthetized with ketamine (140 mg/kg, ip) and decapitated, and the brain was quickly placed into ice-cold artificial cerebrospinal fluid (ACSF) containing (in mM) 124 NaCl, 3 KCl, 1.23 NaH_2_PO_4_, 1.2 MgSO_4_, 26 NaHCO_3_, 10 dextrose and 2.5 CaCl_2_ bubbled continuously with 95% O_2_/5% CO_2_. Horizontal olfactory bulb slices (300 µm thick) were prepared with a vibrating blade microtome (WPI Inc., USA). Fresh slices were incubated in the chamber with carbogenated ACSF and recovered at 30°C for 30 min and then maintained at room temperature.

### Electrophysiological studies

Conventional whole-cell recordings in the current-clamp mode were made with patch pipettes containing (in mM) 140 K-methylsulfate, 4 NaCl, 10 HEPES, 0.2 EGTA, 4 MgATP, 0.3 Na_3_GTP, and 10 phosphocreatine. The pH was adjusted to 7.4 with KOH. The micropipettes were made from borosilicate glass capillaries (Sutter Instrument Co.) and had resistances in the range of 5–8 MΩ. The cells were viewed under an upright microscopy (Eclipse E600-FN, Nomarsky, Nikon Corp., Tokyo, Japan) and recorded with an Axopatch-200B amplifier (Molecular Devices, Palo Alto, CA). Moreover, the MCs were identified by their morphology, size and location [23], [26]. In the GCs recordings, the cells were selected from the granule cell layer (GCL) based on their small cell-body diameters (<10 µm) [27]. The olfactory bulb slices were perfused with 32°C ACSF that was bubbled continuously with carbogen (95% O_2_/5% CO_2_). The EPSPs or inhibitory postsynaptic potentials (IPSPs) were recorded in the control ACSF in the absence of any receptor blockers to ensure that both the glutamatergic and GABAergic neurotransmission were intact. The membrane potentials (mV) of the MCs in the current-clamp mode were from −52 to −61 mV. A bipolar stimulating electrode (inside diameter, 25 µm, FHC Co., USA) was placed into the external plexiform layer (EPL) or granule cell layer (GCL), to evoke synaptic responses at the distal or proximal inputs, respectively. The current intensity of the test stimuli (0.01–0.30 mA) was set to produce half-maximal EPSPs (one-peak monosynaptic responses, with amplitudes between 1 and 5 mV). The basal evoked synaptic responses were produced by 100 µs electrical stimulation at 0.05 Hz except during the induction of STDP. The TBS consists of five bursts of five stimulations (intra bursts: 100 Hz; inter-burst 5 Hz) repeated 5 times at 0.1 Hz. The data were low-pass filtered at 2 kHz and acquired at 5–10 kHz. The series resistance was always monitored during the recording for fear that re-sealing of the ruptured membrane would cause changes in both the kinetics and amplitude of the EPSPs. The cells in which the resistance or capacitance deviated by >20% from the initial values were excluded from the analysis. The data were collected with the pClamp9.2 software and analyzed using Clampfit9.2 (Molecular Devices, Palo Alto, CA).

The STDP in the MCs was induced by pairing the EPSPs with postsynaptic spike bursts. The pairings were repeated 60 times at a 0.1 Hz stimulation and the EPSPs were evoked by stimulating the glomeruli that correlated with the recorded MCs. These postsynaptic bursts comprised of three spikes and were elicited by a current injection (intensity: 50–300 pA; duration: 50 ms). The inter-spike interval (ISI) in control was set as 19–21 ms (20.1±0.2 ms, n = 12; frequency approximately 50 Hz) by adjusting the intensity of the injected current. The time interval Δ*t* was defined as the time between the onset of the compound EPSP and the onset of the action potential (AP) burst (first AP in the burst). The positive time window was defined as the EPSPs that occurred before the postsynaptic bursts, whereas the negative time window referred to the postsynaptic bursts that occurred before the EPSPs. The persistent potentiation or depression was defined as the percentage changes in EPSP or IPSP amplitude during the last 10 min of the recording after the repetitive stimulation. To examine whether direct activation of M/T cells also contribute to the changes in STDP, we usually used one or two single stimuli to EPL or GCL before TBS was delivered and examine whether EPSP can be elicited. In most of our recordings, TBS in EPL or GCL with moderate intensity usually could not directly activate M/T cells. Occasionally we observe EPSPs were induced in MCs. We discarded these cells and did not continue to perform further recording on these cells.

The data are presented as the mean ± SEM. Paired Student's *t* tests were applied as statistical tests if not indicated otherwise, and the statistical significance was asserted for *p*<0.05.Within-group comparisons were performed using a two-tailed *t* test, and the difference between the groups was compared using ANOVA post hoc comparisons. An ANOVA *post hoc LSD* test was used when equal variances were assumed. The differences were considered significant when *p*<0.05 and significance for homogeneity of the variance test was set at 0.1.

## Results

### Distinct long-term plasticity of excitatory inputs to the GCs

**Figure 1:**
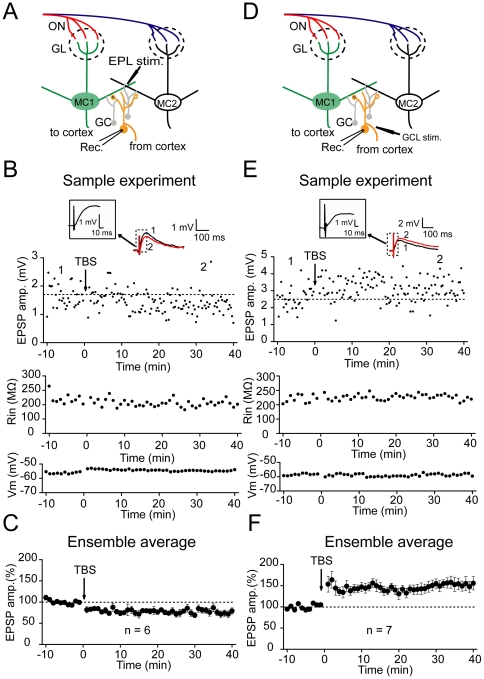
Distinct long-term plasticity of distal and proximal excitatory inputs to GCs. (A) Schematic diagram illustrating a patch recording from a GC when a TBS was delivered on to distal excitatory inputs at the EPL. (B) LTD of distal EPSPs induced by a TBS at the EPL. Representative traces above the graph show changes in the averaged EPSPs selected at the time-points indicated by the number on the graph. The insets in the box represent the enlarged segments of sample traces showing the initial onsets and rising period of the currents. The dashed line indicates the average EPSP amplitude before the TBS. The TBS did not obviously affect the membrane voltage potential (V_m_, middle) or input resistance (R_in_, bottom). The persistent potentiation or depression was defined as the percentage changes in the EPSP amplitude during the last 10 min of recording after the TBS. (C) Summary of changes in the EPSP amplitude following the TBS at distal excitatory inputs at the EPL. (D) Schematic diagram illustrating a patch recording from a granule cell when the TBS was delivered on to proximal excitatory inputs at GCL. (E) An LTP of proximal EPSPs induced by TBS at the GCL. (F) Summary of changes in the EPSP amplitude following a TBS at the proximal excitatory inputs at the GCL (n = 7, *p*<0.001).

In order to investigate whether persistent modification of the inhibitory inputs regulates the predisposition of the subsequent STDP induction of excitatory ON inputs to the MCs, we need to induce both long-term plasticity of inhibitory inputs and STDP of excitatory ON inputs to the MCs. Since GCs are the main type of interneuron in the olfactory bulb that laterally inhibits the M/T cells, we first tried to induce long-term plasticity of excitatory inputs to the GCs and then determine whether this plasticity could in turn elicit long-term plasticity of inhibitory inputs to MCs. We performed whole-cell patch clamp recordings of evoked EPSPs in the GCs of MOB slices to examine whether long-term plasticity of excitatory inputs onto GCs (excitatory inputs→GCs synapses) could be elicited using a specific induction protocol (Fig. 1A). A TBS protocol (five 100-Hz bursts of 5 shocks, repeated at 5 Hz) was focally delivered to the distal or proximal excitatory inputs to the GCs to induce long-term plasticity of these inputs. The EPSPs were recorded in the current clamp mode without disturbing GABA_A_ receptor-mediated inhibitory synaptic transmission. The bipolar stimulating electrode was placed in either the EPL or GCL within a 200–300 µm distance from the cell body of the MCs to stimulate distal or proximal excitatory inputs, respectively (Fig. 1A,D) [28]. Strowbridge and colleagues had employed a two-photon guided minimal stimulation to selectively activate the two inputs [13]. In order to ensure that we also activated a relatively homogenous population of presynaptic processes when stimulating either of the two sites (i.e., the specificity of the stimulations), we deliberately controlled the intensity of the local stimulation to ensure the independence of the stimulation on distinct sites. The synaptic responses we obtained from these two inputs displayed distinct properties in their current kinetics (rise time, GCL: 19.9±3.7 ms, EPL: 44.5±5.2 ms, *p*<0.001, Fig. S1B), similar to the observations obtained from the Strowbridge lab [13]. At the distal excitatory inputs in EPL that mediated local DDI, TBS elicited LTD of these inputs in 6 out of the 7 cells (78.0±2.3%, n = 6, *p*<0.001, paired-samples t test, *p*-values are between baseline and the last 10 minutes after induction; Fig. 1B, C). In contrast, the same protocol, when delivered to the GCL, produced a LTP of the proximal excitatory inputs in 7 out of the 8 cells (153.2±4.1%, n = 7, *p*<0.001, paired-samples t test; Fig. 1E, F). These two forms of synaptic plasticity were not due to a rundown of the EPSPs, because no persistent changes in the EPSP amplitude were observed when the TBS was absent (104.7±6.3%, n = 6, *p*>0.05, Fig. S2). In addition, the synaptic plasticity was not associated with any obvious changes in the input resistance or membrane potential (Fig. S3). The resting potentials of the GCs detected in this study (approximately −60 mV) were in the normal range of the resting potential (−76 to −54 mV) reported by a previous study [29], indicating that the recorded GCs were healthy. Thus, distinct long-term plasticity of excitatory inputs to the GCs could be induced with identical TBS delivered to EPL or GCL.

### Modulation of inhibition onto the MCs by plasticity of the GCs

**Figure 2:**
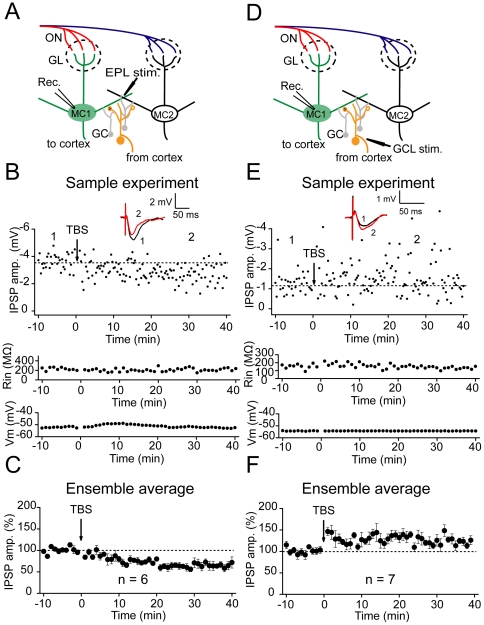
Distinct long-term plasticity of lateral inhibition onto MCs. (A) Schematic diagram illustrating a patch recording from a MC when a TBS was delivered on to distal excitatory inputs at the EPL. (B) LTD of distal IPSPs induced by TBS at the EPL. Representative traces above the graph show changes in averaged IPSPs selected at the time-points indicated by the number on the graph. The TBS did not obviously affect the membrane voltage potential (V_m_, middle) or input resistance (R_in_, bottom). (C) Summary of the changes in IPSP amplitude following TBS at distal excitatory inputs at the EPL (n = 6, *p*<0.001). (D) Schematic diagram illustrating a patch recording from a MC when a TBS was delivered on to the proximal excitatory inputs at the GCL. (E) LTP of proximal IPSPs induced by TBS at the GCL. (F) Summary of changes in the IPSP amplitude following a TBS at proximal excitatory inputs at the GCL (n = 7; *p*<0.001).

Lateral inhibition in the olfactory bulb is largely mediated by reciprocal dendrodendritic synaptic connections between the MC lateral dendrites and the dendrites of inhibitory GCs. It is possible that plasticity at the excitatory inputs onto the GCs may alter their intrinsic property and/or driving force onto the MCs and, consequently, modulate the lateral inhibition onto the MCs. This potential modulation in lateral inhibition might contribute to the refinement of encoding or processing of olfactory information in the MCs. Thus, we further investigated whether the differential plasticity of GC excitatory inputs cause differential long-term plasticity of lateral inhibition in the MCs. The evoked IPSPs were recorded in the MCs by extracellularly stimulating the EPL or GCL (GCs→MCs synapses) before and after TBS (Fig. 2A. 2D). These responses appeared to be mediated by GABA_A_ receptors, as they exhibited a reversed polarity near −70 mV and were blocked by gabazine (10 µM; Fig. S4). We only analyzed the responses from the MCs that showed clear inhibitory responses to single stimulations (see sample traces in Fig. 2B, C). The TBS depressed the IPSPs evoked by the EPL stimulation for at least 30 min (65.7±2.8%, n = 6; *p*<0.001; Fig. 2B,C). Moreover, using the same protocol, the TBS delivered on to the GCL potentiated the IPSPs (124.5±3.3%, n = 7; *p*<0.001; Fig. 2E,F) [13]. These two forms of synaptic plasticity were not associated with obvious changes in the input resistance or membrane potential (Fig. S5). As a control, no persistent changes in the IPSP amplitude were observed when the TBS was absent (95.9±8.7%, n = 4; *p*>0.05; Fig. S6). The resting potentials of the MCs detected in this study (approximately −55 mV) were in the normal range of resting potential (−65 to −47 mV) reported by previous studies [29], [30], indicating that the recorded MCs were healthy. These results revealed that using an identical protocol, which induced plasticity with opposing directions in GCs when delivered to two excitatory GC inputs, could also elicit distinct long-term plasticity of inhibitory inputs onto the MCs.

### STDP of ON inputs

**Figure 3:**
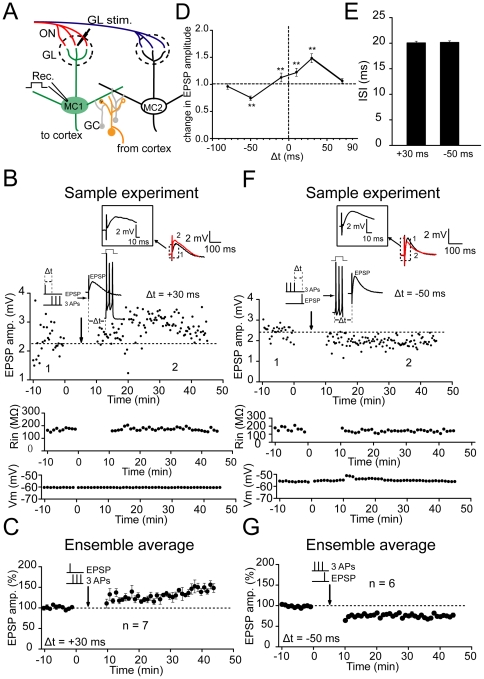
STDP of excitatory olfactory sensory inputs to MCs. (A) Schematic of experimental configuration. Whole-cell recording of a single MC during the application of an extracellular stimulation in an associated glomerulus (the upper dotted line circle) that activated presynaptic excitatory sensory inputs. (B) Potentiation of EPSPs in MCs by a +30 ms pairing (Δt = +30 ms, repeated 60 times). The induction protocol for LTP is depicted on top of the arrow. An EPSP evoked by an extracellular stimulation was paired with a short burst of three APs at 50 Hz elicited by current injections into the postsynaptic cell. The depicted pairing protocol resulted in the potentiation of the EPSP amplitude. The EPSPs averaged at the indicated times are shown on top of the graph. (C) Summary of the changes in the EPSP amplitude following a +30 ms pairing protocol (n = 6; *p*<0.001). (D) STDP plot showing the critical time window for synaptic potentiation and depression of excitatory sensory inputs. The percentage of changes in the EPSP amplitude of synaptic inputs lasting 10 min after the repetitive stimulation was plotted against the time of the inputs (defined by the onset time of the EPSP relative to the onset of the first AP in the burst) [16]. ** *p*<0.01, compared with baseline. (E) Statistical plot showing the ISI when Δt was set to +30 and −50 ms (n = 6), respectively. (F) Depression of EPSPs in MCs by a −50 ms pairing (Δt = −50 ms, repeated 60 times). (G) Summary of the changes in EPSP amplitude following a −50 ms pairing protocol (n = 6; *p*<0.001).

In order to determine whether and how the plasticity of inhibitory inputs mediating lateral inhibition affects STDP of the ON inputs from sensory neurons, we need to further induce STDP in M/T cells. As a Hebbian synaptic learning rule, STDP has been previously demonstrated in various neural circuits, including the olfactory system [48]. Induction protocols for STDP commonly consist of the repetitive pairing of single pre- and postsynaptic spikes at regular intervals. However, neuronal activity in vivo is far more complex [31], with a spectrum of activity level from almost no activity to short bouts of high-frequency spike bursts. Therefore, the pairing of the presynaptic spikes with the postsynaptic bursts sometimes represents a more natural situation in vivo. The lateral inhibition from the GCs may prevent or delay firing or may decrease the firing rate during stimulus presentation in MCs [14], [15]. Because STDP is a form of synaptic plasticity whose induction is heavily dependent on the timing of the incoming spikes and the frequency of the postsynaptic spikes [16], the presence of lateral inhibition in the postsynaptic MCs may profoundly influence the STDP induction and the total olfactory bulb output. We initially found that the repetitive pairing of the EPSPs with single spikes at 0.1 Hz failed to induce any persistent changes in the EPSPs of the ON inputs in MCs (100.4±7.0%, n = 7; *p*>0.05, Fig. S7A; 108.5±6.8%, n = 7; *p*>0.05, Fig. S7). In contrast, the repetitive pairing of the EPSPs with postsynaptic bursts consisting of three postsynaptic APs within a critical time window induced persistent modifications in the EPSPs in MCs (at ON inputs→MCs synapses; Fig. 3). The EPSPs were evoked by stimulating the glomeruli associated with the recorded MCs [32], [33] and could be blocked by co-application of NMDA- and AMPA-type glutamate receptors antagonists such as AP5 (50 µM) and NBQX (20 µM), indicating that they were mediated by glutamatergic neurotransmission (Fig. S4). The postsynaptic APs were elicited by a current injection. The basal level of ISI of the postsynaptic bursts was set to 19–21 ms (20.0±0.3 ms when Δ*t* = +30 ms and 20.1±0.3 ms when Δ*t* = −50 ms; see Fig. 3E) by adjusting the intensity of the injected current. The Δ*t* was defined as the time between the onset of the EPSP and the onset of the first AP in the burst [16], [34]. The pairings were repeated 60 times at 0.1 Hz. We found that the EPSPs potentiated in 6 out of the 8 cells when the Δ*t* was set to +30 ms (143.7±2.5%, n = 6; *p*<0.001; Fig. 3B, C), whereas it was depressed when the Δ*t* was set at −50 ms (74.3±1.3%, n = 6; *p*<0.001; Fig. 3F, G). These two forms of synaptic plasticity were not associated with obvious changes in the input resistance or membrane potential (Fig. S8). The entire time window for the induction of potentiation and depression of excitatory ON inputs was then determined (Fig. 3D). These results indicate that STDP of excitatory OSN inputs to MCs could be induced by pairing EPSPs with postsynaptic spike bursts within a critical time window.

It has been reported that stimulation of ON induced long-lasting depolarizations (LLDs) that lasted as long as several seconds [30], [35], [36], [37]. The LLDs may only occur synchronously in cells whose apical dendrites ramify within the same glomerulus, suggesting that the LLDs involved intraglomerular interactions among the M/T cells. Therefore, glomeruli stimulation in this study might also induce LLDs in the correlated MCs. Indeed, occasionally we observed LLD-like responses in some of our results (Fig. S9). These responses were initiated by a fast and graded monosynaptic input from the OSNs followed by a slower component. To ensure that the EPSPs we detected were mediated by monosynaptic neurotransmission, we discarded those EPSPs with multiple peaks and only used those that displayed single-peak responses. In addition, because ON stimulation with high intensities more frequently produces LLDs, we deliberately controlled the stimulation intensity within moderate level in most of our recordings. Thus, the responses we report here represent monosynaptic inputs to the MCs.

### Regulation of plasticity in the ON inputs

**Figure 4:**
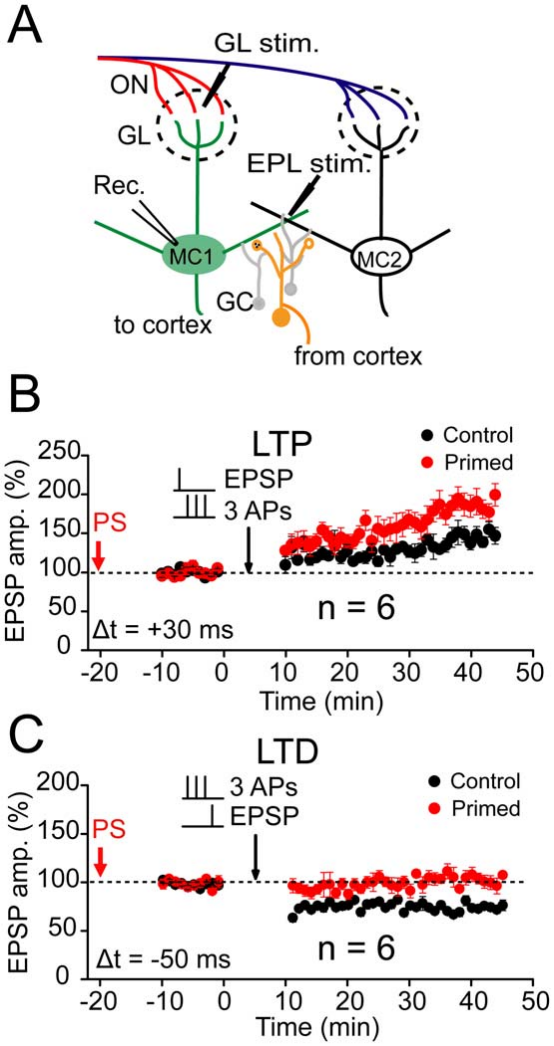
Regulation of STDP by prior TBS of distal inputs at the EPL. (A) Schematic of experimental configuration. Whole-cell recording was conducted in a MC. Bipolar stimulation electrodes were placed at the correlated glomerulus (upper dotted line circle) and the EPL to activate presynaptic excitatory sensory inputs to MCs and distal inputs to GCs, respectively. (B) Prior TBS (PS) of distal inputs at the EPL facilitated LTP by increasing the magnitude of potentiation. Potentiation of EPSPs in MCs was induced with a +30 ms pairing protocol (n = 6; *p*<0.001). The stable EPSP baseline recording was performed 10 min after the end of the previous TBS. (C) PS of distal inputs at the EPL suppressed LTD by decreasing the magnitude of depression (n = 6 ; *p*<0.001). The control data in B and C are the same as in Fig. 3C and G, respectively.

**Figure 5:**
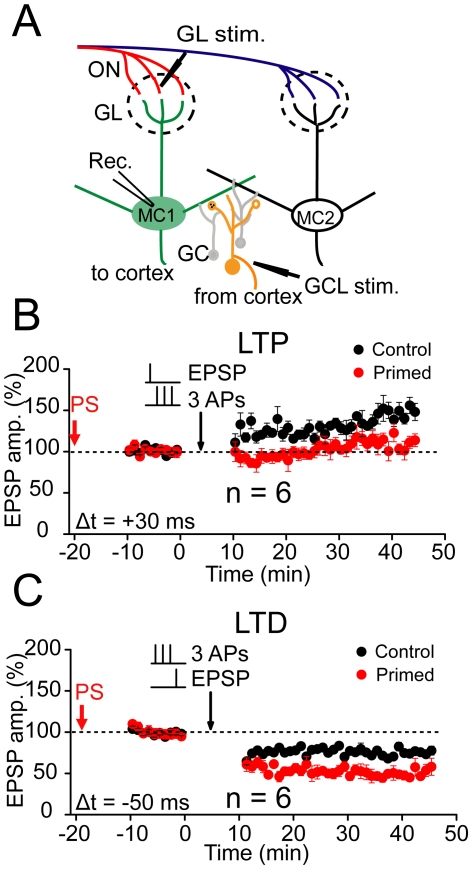
Regulation of STDP by prior TBS of proximal inputs at the GCL. (A) Schematic of experimental configuration. Bipolar stimulation electrodes were placed at a correlated glomerulus (upper dotted line circle) and GCL to activate presynaptic excitatory sensory inputs to MCs and proximal inputs to GCs, respectively. (B) PS of proximal inputs at the GCL suppressed LTP by decreasing the magnitude of potentiation (n = 6; *p*<0.001). The potentiation of EPSPs in MCs was induced with a +30 ms pairing protocol. The stable EPSP baseline recording was performed 10 min after the end of the previous TBS. (C) Prior TBS of proximal inputs at the GCL facilitated LTD by increasing the magnitude of depression (n = 6; *p*<0.001). The control data in B and C are the same as in Fig. 3C and G, respectively.

After confirming the feasibility of the STDP induction, we further investigated whether and how the prior plasticity of lateral inhibition affects the subsequent STDP of ON inputs from the OSNs. We began to collect whole-cell baseline EPSP data in MCs approximately 10 min after the prior stimulation was finished (Fig. 4A). The baseline EPSP recording in MCs lasted for 10 min and was followed by a STDP induction. Thus, the interval between the end of the prior TBS stimulation and subsequent STDP induction was 20 min (Fig. 4B, C). The prior TBS protocol, which was used to induce LTP or LTD of lateral inhibition when delivered to the proximal or distal inputs to GCs, respectively, did not affect the baseline EPSPs of ON inputs in MCs (OSN→MCs synapses; 106.8±3.2%, n = 6; *p*>0.05, Fig. S10B; 96.7±1.9%, n = 6, *p*>0.05; Fig. S10D). However, it modified the predisposition for the subsequent spike timing-dependent LTP or LTD induction in MCs. The protocol used in EPL to induce the LTD of lateral inhibition facilitated the subsequent LTP of EPSPs on ON inputs by elevating the magnitude of potentiation (186.0±2.4%, n = 6; compared with control, *p*<0.001; Independent-samples t test; Fig. 4B), whereas it suppressed LTD by reducing the magnitude of depression (99.8±1.2%, n = 6; compared with control, *p*<0.001; Independent-samples t test; Fig. 4C). In contrast, when the same stimulating protocol was delivered to the proximal inputs onto the GCs to induce LTP of lateral inhibition (Fig. 5A), it suppressed the subsequent LTP of EPSPs on ON inputs by reducing the magnitude of potentiation in MCs (109.1±2. 2%, n = 6; compared with control *p*<0.001; Independent-samples t test; Fig. 5B), whereas it facilitated LTD by elevating the magnitude of depression (50.1±1.5%, n = 6; compared with control, *p*<0.001; Independent-samples t test; Fig. 5C). These results reveal the bidirectional regulation of STDP by prior TBS of dictinct inputs onto GCs and suggest the changes in STDP may be caused by plasticity originated in GCs.

If the changes in STDP are truly caused by plasticity originated in GCs, then blockade of this plasticity should also suppress the changes in STDP. To test this possibility, we treated the slices with antagonists of AMPA- and NMDA-type glutamate receptor only during TBS to block the glutmatergic neurotransmission. This treatment totally abolished the induction of plasticity in GCs. (EPL 94.0±2.1%, n = 5; compared with baseline *p*>0.05; Fig. S11A; GCL 91.6±3.5%, n = 4; compared with baseline *p*>0.05; Fig. S11C). As a result, no subsequent changes in STDP were observed (EPL 132.9±8.0%, n = 6; compared with control *p*>0.05; Fig. S11B; GCL 140.2±5.3%, n = 7; compared with control *p*>0.05; ANOVA *LSD* test; Fig. S11D). Taken together, these data strongly suggest that the prior plasticity history of lateral inhibition driven by the two distinct excitatory inputs to GCs exerted differential regulation on the subsequent plasticity of excitatory inputs from the OSNs.

### Changes in the ISI is the causative factor for regulation of plasticity

**Figure 6:**
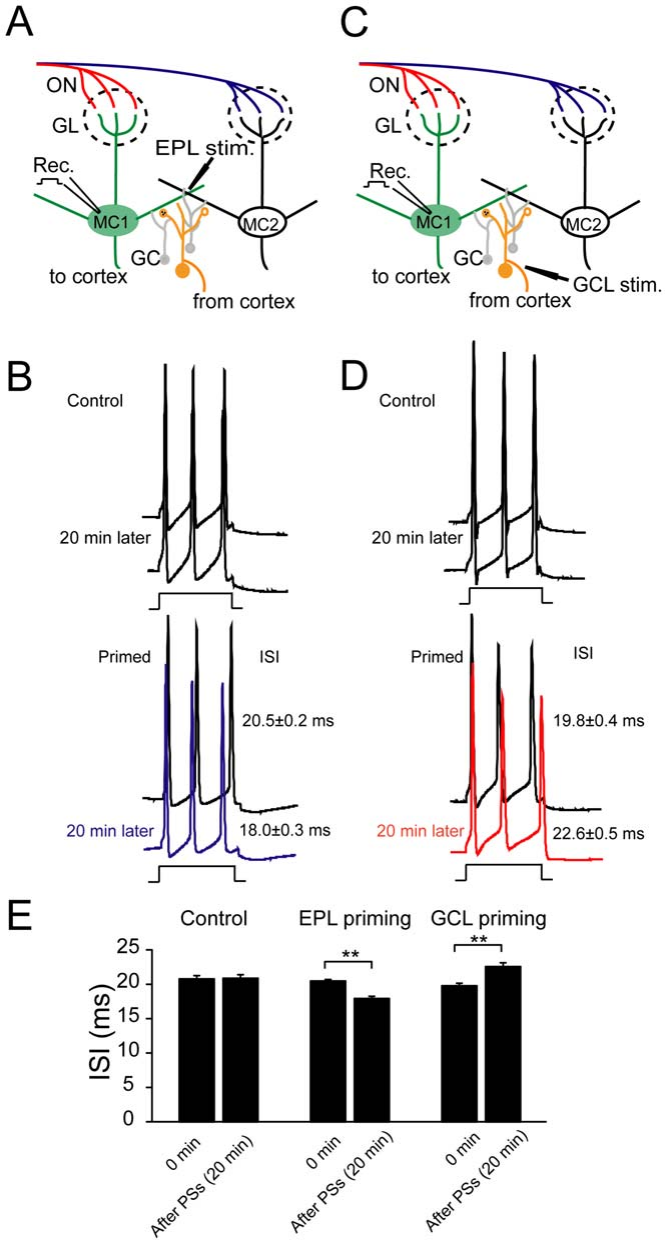
PS at distal or proximal inputs bidirectionally regulate the ISI of spike bursts. (A) Schematic of experimental configuration. The TBS was delivered to distal inputs to GCs in the EPL. (B) Representative traces of spike bursts evoked by current injections under conditions with or without prior stimulation. Top, without prior stimulation, the ISI of spike bursts failed to display any obvious changes 20 min later (control). Bottom, ISI was shortened 20 min after prior stimulation at distal inputs to GCs (primed), which was at the exact time-point of STDP induction. (C) Schematic of the experimental configuration. The TBS was delivered to proximal inputs to GCs at the GCL. (D) Representative traces of spike bursts evoked by current injection under conditions with or without prior stimulation. Bottom, the ISI was prolonged 20 min after prior stimulation at proximal inputs to GCs. (E) Summary of changes in the ISI of spike bursts under various conditions. ** *p*<0.01, compared between indicated groups.

What is the mechanism that underlies the regulation of plasticity in ON inputs? It has been reported that recent activity history could affect the lateral inhibition between MCs and the lateral inhibition may alter MC spike-timing [15]. In addition, the frequency of postsynaptic spikes within the pairing protocol can affect the induction and magnitude of STDP [16], most likely due to a change in the back propagation of spike trains [38], [39]. This raises the possibility that the regulation of STDP exerted by prior plasticity in lateral inhibition was achieved by regulating the ISI or frequency of APs in the burst. Therefore, we further examined in MCs whether and how the prior stimulating protocol affected the subsequent spike patterns within the induction protocol for STDP. Here, the TBS on the EPL or GCL was defined as the priming stimulation and the recordings were performed on the MCs. Interestingly, we found that prior EPL stimulation of distal inputs onto the GCs significantly shortened the ISI of the three-spike-burst at the time-point when the subsequent pairing protocol was delivered (ISI 18.0±0.3 ms, n = 8; compared with baseline 20.5±0.2 ms, *p*<0.001; paired-samples t test; Fig. 6B, E). In contrast, an identical prior stimulating protocol delivered to GCL prolonged the ISI (22.6±0.5 ms, n = 7, compared with baseline, *p*<0.001; Fig. 6B, E). As a control, the ISI of the spike burst was kept unchanged when no prior stimulation was delivered (20.9±0.5 ms n = 6, compared with baseline 20.8±0.5 ms, *p*>0.05; Fig. 6B, D, E). These results suggested that the regulation of the subsequent STDP may be correlated with coincident changes in the ISI within the STDP induction protocol.

**Figure 7:**
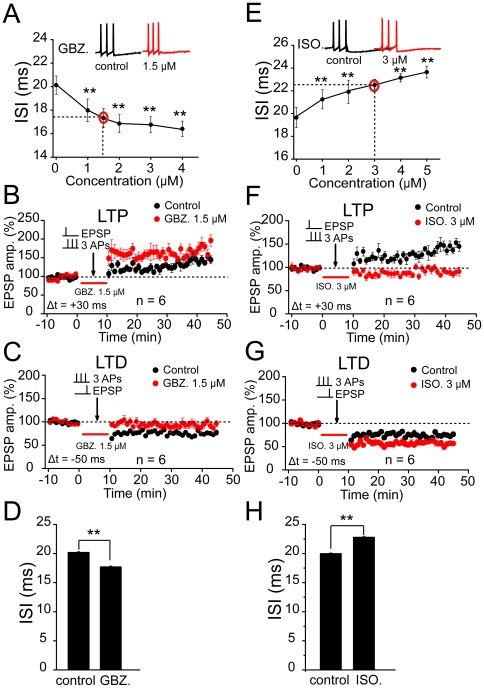
Pharmalogical manipulation of ISI mimicked the regulation of STDP by prior TBS. (A) Dose-response relationship of a selective GABA receptor antagonist Gabazine (GBZ). Sample traces at the top, GBZ at a concentration of 1.5 µM shortened the ISI similarly to the prior EPL stimulation. (B) Application of 1.5 µM GBZ during STDP induction facilitated LTP by increasing the potentiation magnitude (n = 6, *p*<0.001). (C) Application of 1.5 µM GBZ during STDP induction suppressed LTD by decreasing depression magnitude (n = 6, *p*<0.05). (D) Statistical plot showing the effect of GBZ (1.5 µM) in shortening the ISI (n = 6, *p*<0.01). (E) Dose-response relationship of a selective GABA receptor agonist isoguvacine (ISO). Sample traces at the top, ISO at a concentration of 3.0 µM prolonged the ISI similarly to the prior GCL stimulation. (F) Application of 3.0 µM ISO during STDP induction suppressed LTP by decreasing the potentiation magnitude (n = 6, *p*<0.01). (G) Application of 3.0 µM ISO during STDP induction facilitated LTD by increasing the depression magnitude (n = 6, *p*<0.001). (H) Statistical plot showing the effects of ISO (3.0 µM) in prolonging the ISI (n = 6, *p*<0.01). The control in B and F were same as those in Fig. 3C, and the control in C and G were obtained from Fig. 3G.

Although these results suggest that the changes in the ISI and the subsequent regulation of LTP/LTD may be correlated, we still do not know whether the changes in the ISI are the causative factor for the regulation of STDP. It has been reported that partially up- or down-regulating GABA_A_ receptor function with its agonist or antagonist can bidirectionally regulate the ISI within a spike burst [40], [41], [42]. Therefore, to examine this possibility, we used a selective GABA_A_ receptor agonist or antagonist to determine how it affected the pattern of spikes within the induction protocol. Gabazine (GBZ) and isoguvacine (ISO) are highly selective GABA_A_ receptor antagonists and agonists, respectively. We first investigated the dose-response relationship of these two agents and determined the concentration that matched the ISI regulation exerted by the previous stimulating protocol. We found that a partial blockade of GABA_A_ receptor function with GBZ at 1.5 µM displayed similar effects on the ISI exerted by a prior stimulation at the EPL (Fig. 7A), whereas a submaximal activation of GABA_A_ receptor function with ISO at 3.0 µM produced comparable effects on the ISI exerted by a prior stimulation at the GCL (Fig. 7E). This partial manipulation of GABA_A_ receptor function could mimic, to a certain extent, the prior potentiation or depression of lateral inhibition the MCs received from the TBS. It was reported that the GABA_A_ receptor blockade increased the excitatory MC responses to odors [21], suppressed the adaptation of MC firing rate [15] and disrupted the animal's ability to distinguish between similar odors [43]. However, the concentration of drugs used in this study were largely decreased compared with previous studies [40], [44], and we determined that GBZ at this level failed to clearly or persistently affect the cell excitability, manifested by the absence of changes in the membrane potential or synaptic responses (Fig. S12; n = 4, ANOVA *LSD* test, *p*>0.05). Thus, we largely avoided these unwanted side effects. Then we examined how these treatments could affect the induction of the STDP. GBZ (1.5 µM) facilitated spike timing-dependent LTP (tLTP) by increasing the magnitude of potentiation at OSN→MCs synapses (172.3±4.7%, n = 6, *p*<0.001, Independent-samples t test; Fig. 7B), but suppressed spike timing-dependent LTD (tLTD) by decreasing the magnitude of depression (93.6±1.5%, n = 6, *p*<0.001; Fig. 7C). In contrast, the ISO at 3.0 µM suppressed tLTP induction by decreasing the magnitude of potentiation (92.9±1.5%, n = 6, *p*<0.001; Fig. 7F), but facilitated tLTD by increasing the magnitude of depression (58.8±1.2%, n = 6, *p*<0.001; Fig. 7G). These results demonstrate that mimicking ISI modification with a GABA_A_ receptor agonist or antagonist could cause a similar modification of the predisposition for subsequent STDP induction. To further ensure that the change in the ISI was the causative factor for plasticity modification, we injected three shorter current steps to the recorded MCs so that each current step was sufficiently strong to trigger a single action potential. When we set the ISI to 17.4 ms and 22.9 ms to mimic the bidirectional changes in the ISI following the prior stimulations, we were able to observe similar changes in the STDP (192.4±3.2%, n = 6, compared with control: 165.5±15.4% *P*<0.001, Fig. S13B; 97.7±2.0%, n = 6, compared with control: 78.8±7.4% *P*<0.001, Fig. S13C; 95.8±2.4%, n = 6, compared with control: 165.5±15.4% *P*<0.001, Fig. S13E; 61.3±1.0%, n = 6, compared with control: 78.8±7.4% *P*<0.001, Fig. S13F). These results suggest that the changes in ISI may contribute to the regulation of STDP.

The regulation of the ISI that was present continually after the TBS did not require acute GC stimulation, suggesting that TBS causes a tonic change in inhibition. To further examine whether the regulation of the ISI depend on a tonic change in inhibition that was continuously present after TBS, we also monitored miniature IPSPs (mIPSPs) before and after TBS and found that the TBS at EPL suppressed the mIPSPs amplitude in MCs (control, 1.68±0.15 mV, after EPL TBS, 1.38±0.09 mV; *p*<0.01; Fig. S14). These results provided further evidence that the TBS induces tonic and continuous regulation of the ISI and suggest that the changes in the ISI by prior plasticity of lateral inhibition may be the causative factor for the regulation of STDP. Moreover, it also suggests that modification in the GC-to-MC synapse may take a role in changes of inhibition.

**Figure 8:**
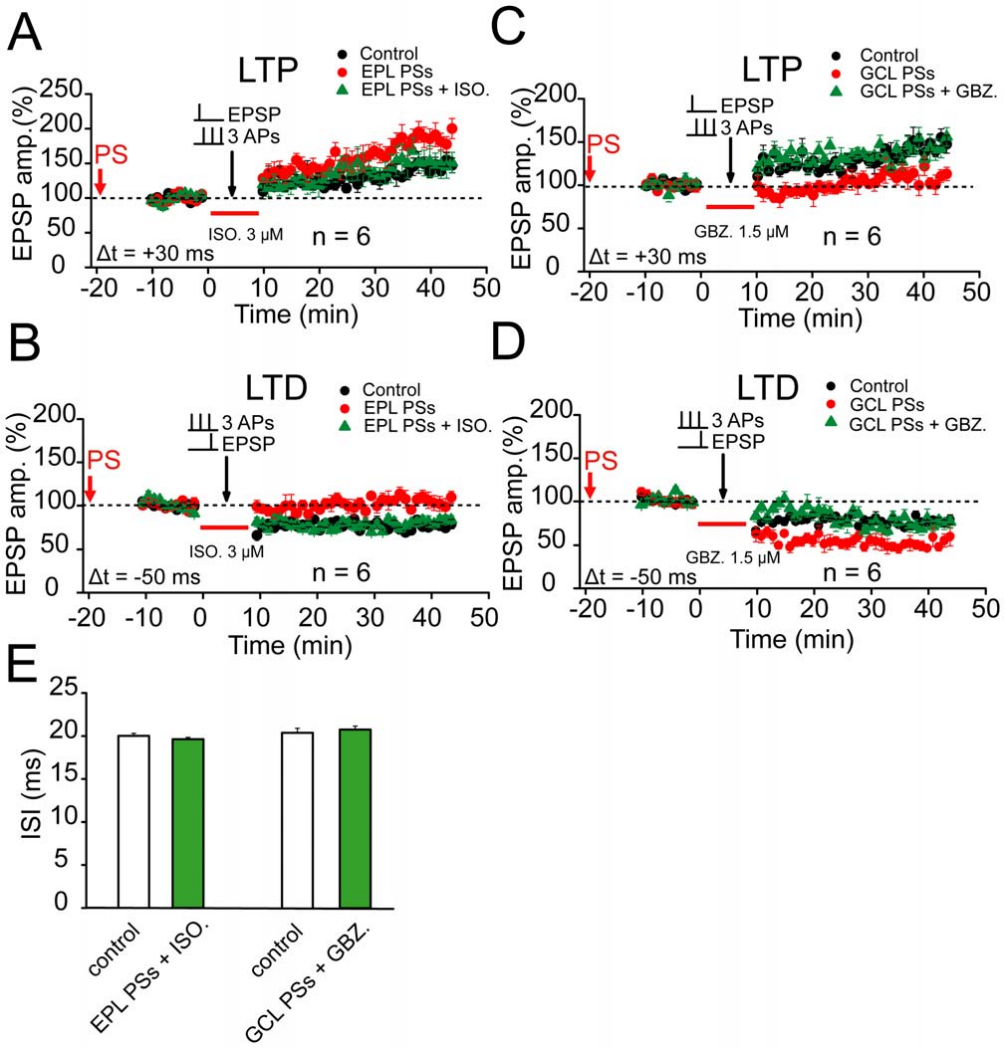
Bidirectional regulation of STDP. (A) The effects of prior EPL stimulation on the subsequent LTP induction were reversed by the interleaved application of ISO (3.0 µM). The PS alone delivered to distal inputs at the EPL (EPL PSs) led to the enhancement of the LTP magnitude produced with a +30 ms pairing protocol (Δt = +30 ms). However, this protocol failed to facilitate LTP after the application of ISO during the STDP induction (EPL PSs+ISO; n = 6, compare with EPL PSs, *p*<0.01, compared with baseline, *p*>0.05). The arrow refers to the time-point of PS. (B) The effect of prior EPL stimulation on subsequent LTD induction was reversed by the interleaved application of ISO (3.0 µM; EPL PSs+ISO; n = 6, compare with EPL PSs, *p*<0.01, compared with control, *p*>0.05). The LTD was induced with a −50 ms pairing protocol (Δt = −50 ms). (C) An interleaved application of GBZ (1.5 µM) reversed the effect of the prior GCL stimulation on the subsequent LTP induction. Prior TBS alone delivered to proximal inputs at the GCL (GCL PSs) led to the suppression of the LTP magnitude produced with a +30 ms pairing protocol. An interleaved application of GBZ (1.5 µM; GCL PSs+GBZ) during STDP induction reversed the suppression of LTP by the GCL PSs (n = 6; *P*<0.01). (D) An interleaved application of GBZ (1.5 µM) reversed the effect of prior GCL stimulation on the subsequent LTD induction (n = 6, *p*<0.01). The control in A and C were the same as those in Fig. 3C, and the control in B and D were obtained from Fig. 3G. (E) Statistical plot showing that ISO (n = 6) or GBZ (n = 6) application could reverse the effects of EPL and GCL stimulation on the ISI to control the no-priming level (*p*<0.01).

If the changes in ISI really contribute to the regulation of STDP, then reversing the changes in ISI by resetting ISI back to control level should also abolish changes in STDP. To this purpose, we either employed pharmacological intervention of ISI with GABA_A_ agonist/antagonist or directly reset the ISI with the three individual APs to get the ISI back to control levels (around 20 ms) after TBS. Following the EPL priming stimulation, which induced LTD of lateral inhibition, we applied 3.0 µM of ISO immediately before and during the pairing stimulation protocol for STDP induction. This treatment elevated the ISI value in MCs, which was already decreased by the prior EPL stimulation, to no-priming control levels (19.6±0.2 ms, n = 6, compared with control, 20.0±0.3 ms, *p*>0.05; Fig. 8E). As a result, the induction with a pairing protocol failed to elicit any persistent changes in the magnitude of spike timing-dependent LTP or LTD at OSN→MCs synapses, similar to controls without prior stimulation (LTP 152.9±1.9%, n = 6, *p*>0.05, ANOVA *LSD* test; LTD 77.9±1.4%, n = 6, *p*>0.05; Fig. 8A, B). Similarly, treating with 1.5 µM GBZ following GCL priming stimulation decreased the ISI value, which was already increased by prior GCL stimulation, to no-priming control levels (20.8±0.4 ms, n = 6, compared with control 20.4±0.5 ms, *p*>0.05; Fig. 8E). As a result, the induction with the pairing protocol failed to elicit any persistent changes in the magnitude of LTP or LTD (LTP 141.7±2.9%, n = 6, *p*>0.05, ANOVA *LSD* test; LTD 73.2±1.4%, n = 6, *p*>0.05; Fig. 8C, D). Moreover, adjusting the current injection level after prior TBS to convert the ISI back to control levels also reversed the STDP to control levels (147.5±1.9%, compared with control, *p*>0.05, Fig. S15A; 145.2±1.1%, compared with control, *p*>0.05, Fig. S15B). Taken together, these results further confirm that the change in the ISI is the causative factor for regulation of plasticity.

## Discussion

In the olfactory system, few studies have investigated the regulation of plasticity in olfactory sensory input by prior activity from GCs. This study demonstrates that using an identical TBS stimulation delivered to the distal or proximal inputs could induce persistent modification in the inhibition to MCs with opposing directions. This bidirectional modification of the inhibitory inputs differentially regulated the subsequent synaptic plasticity of excitatory ON inputs to the MCs. Because the modulation of the inhibition onto the MCs could alter the firing rate of spikes [14], [15], the observed changes in the ISI caused by the plasticity in the excitatory inputs of GCs could be ascribed to several scenarios. First, the differential modification of drives in GCs to MCs is the simplest explanation for the TBS-mediated persistent changes of mitral cell inhibition. Second, the plasticity in the GC-to-MC synapse may also be involved. Third, the regulation of GC spiking by plasticity of excitatory inputs may relieve the Mg^2+^ blockade of NMDA receptors at the dendrodendritic synapses and, thus, dynamically regulate the inhibition of the MCs [13].

The neurons that fire high-frequency bursts of spikes were found in various sensory systems including the olfactory system [14], [45]. The firing of the bursts in response to sensory input depends on the intrinsic cellular mechanisms that function with feedback from higher centers to control the discharge properties of these neurons. A growing number of studies indicate the possibility that the bursts possess a distinct function in the transmission of sensory information. It has been observed that the intrinsic membrane properties favored MCs firing at 40 Hz and the MC discharge was also stabilized at a preferred frequency of 40 Hz [46]. In addition, additional bursts of action potentials may be triggered by the synaptic inputs locked to the air inhalations. Moreover, it has been proposed that neuronal oscillations enhanced stimulus discrimination by ensuring AP precision, and the maintenance of AP precision could be compromised at oscillation frequencies higher than 50 Hz [47]. These findings imply that the natural spike frequency in MCs in vivo that permits high levels of optimal stimulus discrimination is approximately 50 Hz. In this study, the STDP in MCs was induced by the pairing of presynaptic EPSPs with realistic 50 Hz postsynaptic bursts within a critical window of tens of milliseconds. The changes in synaptic strength depend on the inter-burst interval rather than the precise timing of the individual spikes [16]. These burst timing-dependent plasticity rules may be specifically beneficial for the circuits in which the information relevant for synaptic refinement is contained in the timing of the bursts rather than that of the individual spikes. Given the very precise dependence of the magnitude of STDP on the ISI, a slight change in the ISI in MCs may produce significant implications in the output of the olfactory bulb and, thus, may have a particular physiological relevance in the olfactory system.

In the developing olfactory bulb, the inhibitory synapses distributed along the secondary dendrites of the MCs can dynamically regulate the extent of spike propagation, with a smaller activation of the inhibitory synapses facilitating the spike propagation [48]. The lateral and recurrent inhibitions in the olfactory bulb play distinct roles in shaping the MC spiking pattern, which is critical to odor information processing [49]. Both the two forms of inhibition are thought to be important in odor discrimination and in the generation and synchronization of odor-evoked rhythmic MC activity [26], [50], [51]. Although the inhibition we observed in this study is largely driven by the excitatory inputs on to the GCs and thus may largely represent the lateral inhibition, we could not exclude the potential contribution of recurrent inhibition [15].

The two types of anatomically distinct excitatory inputs on to the proximal and distal dendrites of GCs may exert different functions through distinct mechanisms. The excitatory input onto the distal dendrites in the EPL mediates local dendrodendritic inhibition [25], whereas the excitatory inputs onto the proximal dendrites at least partially, if not completely, are cortical feedback inputs that mediate global top-down modification of the DDI. Using a two-photon guided minimal stimulation in acute rat brain slices, Strowbridge and colleagues positioned an extracellular stimulating electrode near a specific dendritic segment and thus were able to activate relatively homogenous populations of presynaptic processes, based on the kinetic properties of the resulting postsynaptic currents [13], [24]. Although we did not use a similar technique, we were able to deliberately control the intensity of the local stimulation to ensure the independence of the stimulation on distinct sites in this study. Moreover, the synaptic responses we obtained from these two inputs also displayed distinct properties in the current kinetics, similar to the findings obtained from Strowbridge's group [13]. Therefore, we could still activate a relatively homogenous population of presynaptic processes when stimulating either of the two sites. Because the extent of GC function in the local versus global output modes could exert a critical effect on the computational role it performs in the olfactory bulb circuit [1], our present findings of the modification of excitatory ON inputs by prior plasticity of the distinct excitatory inputs to GCs reveal an efficient route to regulate the encoding and processing of odor information in the bulb.

## Supporting Information

Figure S1 **Kinetic differences between distal and proximal EPSPs.** (A) Re-scaled and overlaid sample traces showing the difference in the rise time between the responses evoked from the two stimulus positions. (B) A summary plot shows a statistically significant difference in the mean EPSP rise time for the two stimulation sites (EPL, n = 6; GCL, n = 7; ** *p*<0.01).
(TIF)

Figure S2 **No persistent changes in the EPSPs amplitude were observed when TBS was absent.** (A) There was an absence of change in the EPSPs when the TBS was not delivered. Representative traces above the graph show the averaged EPSPs selected at the time-points indicated by the number on the graph. The dashed line indicates the average EPSP amplitude. No obvious changes in the membrane voltage potential (R_in_, middle) or input resistance (V_m_, bottom) were detected. (B) Summary of the averaged data in experiments as shown in A. The EPSP amplitude was evaluated during the last 10 min and is presented as a percentage of the baseline EPSP amplitude. No rundown of the EPSP amplitude was observed (*p*>0.05).
(TIF)

Figure S3 **TBS-induced synaptic plasticity was not associated with obvious changes in the input resistance or membrane potential.** (A) and (B) The statistical profiles of the changes in the averaged input resistance (A; Rin) and membrane potential (B; Vm) associated with the TBS-induced LTD in GCs. (C) and (D) The statistical profiles of the changes in averaged input resistance (C; percentage of baseline) and membrane potential (D) associated with TBS-induced LTP in GCs. No significant changes in the Rin or Vm were detected during the TBS-induced synaptic plasticity in GCs.
(TIF)

Figure S4 **EPSPs and IPSPs recorded in MCs were blocked by an antagonist for glutamate or the GABA_A_ receptor.** (A) The EPSPs recorded in MCs were abolished by co-application of NMDA- and AMPA-type glutamate receptors antagonists AP5 (50 µM) and NBQX (20 µM), whereas the IPSPs were abolished by application of the GABA_A_ receptor antagonist GBZ (10 µM), suggesting that they were mediated by the glutamate receptor and GABA_A_ receptor, respectively.
(TIF)

Figure S5 **TBS-induced synaptic plasticity was not associated with obvious changes in the input resistance or membrane potential.** (A) and (B) The statistical profiles of the changes in the averaged input resistance (A; Rin) and membrane potential (B; Vm) associated with the TBS-induced LTD in MCs. (C) and (D) The statistical profiles of the changes in averaged input resistance (C; percentage of baseline) and membrane potential (D) associated with TBS-induced LTP in MCs. No significant changes in the Rin or Vm were detected during the TBS-induced synaptic plasticity in MCs.
(TIF)

Figure S6 **No persistent changes in the IPSPs amplitude were observed when the TBS was absent.** Absence of changes in the IPSPs when TBS was not delivered. Representative traces above the graph show averaged IPSPs selected at the time-points indicated by the number on the graph. The dashed line indicates the average IPSP amplitude. No obvious changes in the membrane voltage potential (R_in_, middle) or input resistance (V_m_, bottom) were detected. (B) Summary of the averaged data in experiments as shown in A. The IPSP amplitude was evaluated during the last 10 min and normalized to the baseline IPSP amplitude. No rundown of the IPSP amplitude was observed.
(TIF)

Figure S7 **Pairing presynaptic EPSP with single postsynaptic spikes failed to induce any persistent changes in the EPSP in MCs.** (A) When repetitive EPSPs preceded the single postsynaptic action potentials induced by injected currents at a +30 ms time window, no long-lasting changes in the EPSPs were detected. (B) When repetitive single postsynaptic action potentials preceded EPSPs at a −50 ms time window, changes in the EPSPs were absent.
(TIF)

Figure S8 **STDP in MCs was not associated with obvious changes in the input resistance or membrane potential.** (A) and (B) The statistical profiles of the changes in the averaged input resistance (A; Rin) and membrane potential (B; Vm) associated with the spike timing-dependent LTP in MCs. (C) and (D). The statistical profiles of the changes in the averaged input resistance (C) and membrane potential (D) associated with the spike timing-dependent LTD in MCs. No significant changes in Rin or Vm were detected during the TBS-induced synaptic plasticity in MCs.
(TIF)

Figure S9 **A sample trace showing multiple components in one recording of EPSPs in MCs.** The initial peak currents represent monosynaptic responses.
(TIF)

Figure S10 **The prior TBS protocol did not affect baseline EPSPs of olfactory inputs.** (A) and (C) Schematic of the experimental configuration. The TBS was delivered to distal inputs to the GCs at the EPL (A) or proximal inputs at the GCl (C). (B) and (D) Prior TBS at the EPL (B; EPL PSs; n = 6) or at the GCL (D; GCL PSs; n = 6) did not display an obvious effect on the baseline EPSPs of MCs.
(TIF)

Figure S11 **Blocking glutamatergic neurotransmission during TBS abolished plasticity in GCs and reversed the change in STDP in MCs.** (A) and (C) Blocking glutamatergic neurotransmission when TBS was delivered onto distal (A; 94.0±2.1%, n = 5; compared with baseline *p*>0.05) or proximal inputs (C; 91.6±3.5%, n = 4; *p*>0.05) abolished long-term plasticity in GCs. (B) and (D) Absence of Changes in STDP in MCs when plasticity in GCs was abolished by blocking glutamatergic neurotransmission during TBS onto distal (B; 132.9±8.0%, n = 6; compared with control *p*>0.05, ANOVA *LSD* test) or proximal inputs (D; 140.2±5.3%, n = 7; compared with control *p*>0.05).
(TIF)

Figure S12 **Absence of changes in the synaptic responses and membrane potentials following GBZ or ISO application.** (A) GBZ application failed to affect the EPSC amplitude (top) and membrane potentials (V_m_; bottom). Examples on the top show the synaptic responses before and after GBZ application. (B) Summary of the data showing the absence of changes in the EPSP amplitude following GBZ (1.5 µM) treatment. (C) Histogram plot showing the absence of changes in the V_m_ following GBZ treatment (n = 4, *p*>0.05). (D) The ISO (3.0 µM) did not change the amplitude of the EPSPs. (E) Summary of the data showing the absence of changes in the EPSP amplitude following ISO (3.0 µM) treatment. (F) Histogram plot showing the absence of changes in the V_m_ following ISO treatment (n = 4, *p*>0.05).
(TIF)

Figure S13 **Manipulation of the ISI by adjusting the frequency of spikes mimicked regulation of plasticity by prior TBS.** (A) Representative trace of spike bursts evoked by three short current steps with the ISI set at 17.4 ms. (B) Summary of the changes in the EPSP amplitude showing facilitation of the LTP in MCs. When the ISI was set at 17.4 ms by adjusting the frequency of the spikes induced by three short current steps, the repetitive pairing of EPSPs with the bursts at a +30 ms time window (Δt = +30 ms, repeated 60 times) produced a LTP with a greater magnitude (n = 6, *p*<0.001). (C) Summary of changes in the EPSP amplitude showing a suppression of LTD. When the ISI was set at 17.4, the LTD produced by the pairing protocol at a −50 ms time window (Δt = −50 ms, repeated 60 times) was suppressed (n = 6, *p*<0.001). (D) Representative trace of spike bursts evoked by three short current steps with the ISI set at 22.9 ms. (E) Summary of the changes in the EPSP amplitude showing suppression of LTP. When the ISI was set at 22.9 ms, the repetitive pairing of EPSPs with the bursts at a +30 time window produced LTP with a decreased magnitude (n = 6, *p*<0.001). (F) Summary of changes in the EPSP amplitude showing facilitation of LTD. When the ISI was set at 22.9 ms, the LTD with a greater magnitude was produced by the pairing protocol at a −50 ms time window (n = 6, *p*<0.001). Interestingly, the bidirectional manipulations of the ISI, which mimicked the changes in the ISI produced by the PSs, induced a similar modification of the STDP.
(TIF)

Figure S14 **TBS induced tonic inhibition of mIPSPs in MCs.** (A) Sample traces before and after TBS displays the change in IPSP amplitude. (B) Summary of the change in the mIPSP amplitude following TBS at the EPL.
(TIF)

Figure S15 **Bidirectional regulation of STDP by resetting the ISI after prior TBS.** (A) The EPL priming stimulation (EPL PSs) altered the ISI of the burst induced by a single current injection. This change in the ISI could be reversed by regulating the ISI back to control levels via three individual current injections at a frequency similar to the control. As a result, the LTP was reverted back to control levels. (B) A similar observation was made when both the GCL priming stimulation (GCL PSs) and the resetting of the ISI were performed. The data obtained from the control, EPL PSs and GCL PSs in this figure were taken from Fig. 4B and Fig. 5B for comparison.
(TIF)
