# Supplementary figures and images for: Regulation of Spike Timing-Dependent Plasticity of Olfactory Inputs in Mitral Cells in the Rat Olfactory Bulb
Source: PLoS One. 2012 Apr 19;7(4):e35001. doi: 10.1371/journal.pone.0035001 (PMC3334975; doi:10.1371/journal.pone.0035001)

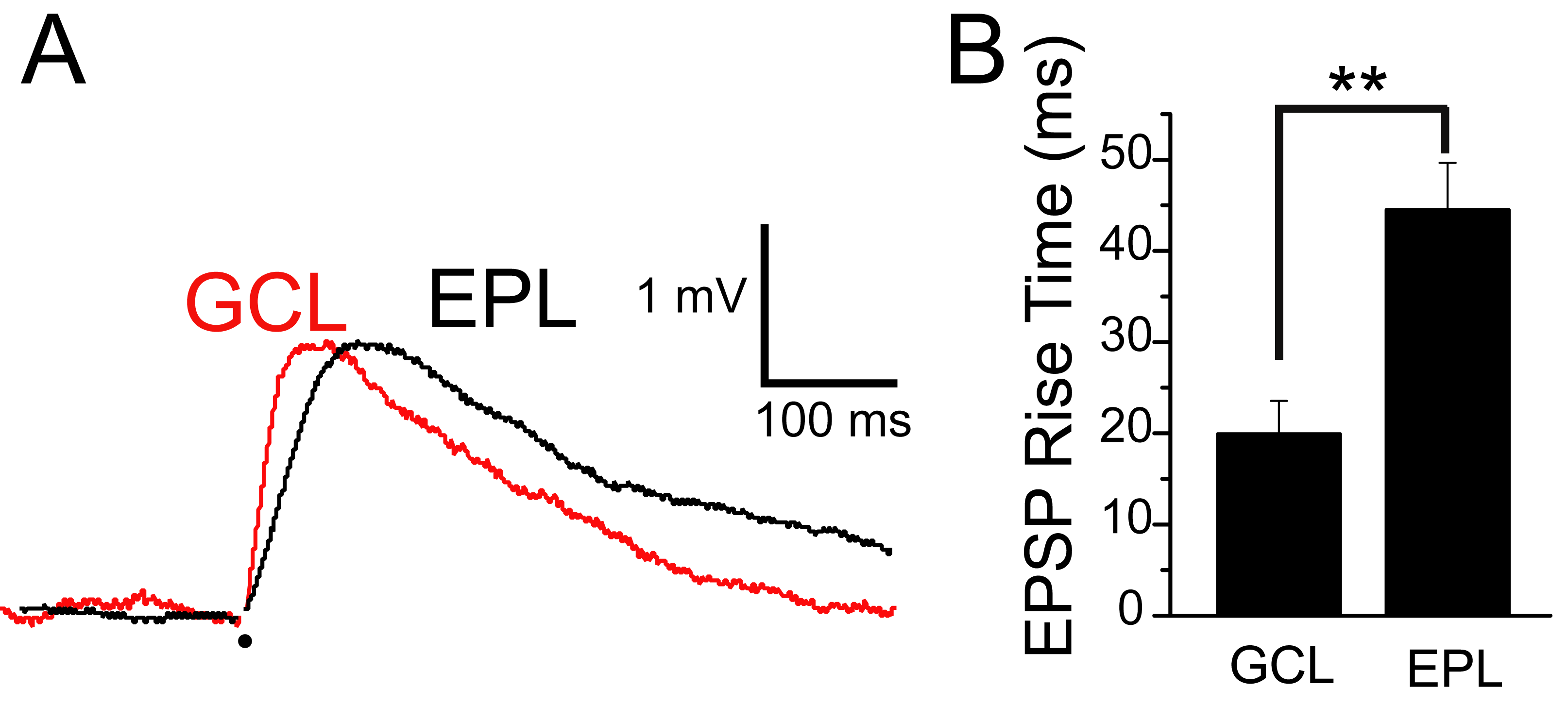

Supplement: Figure S1 — Kinetic differences between distal and proximal EPSPs. (A) Re-scaled and overlaid sample traces showing the difference in the rise time between the responses evoked from the two stimulus positions. (B) A summary plot shows a statistically significant difference in the mean EPSP rise time for the two stimulation sites (EPL, n = 6; GCL, n = 7; ** p<0.01). (TIF) [file pone.0035001.s001.tif]

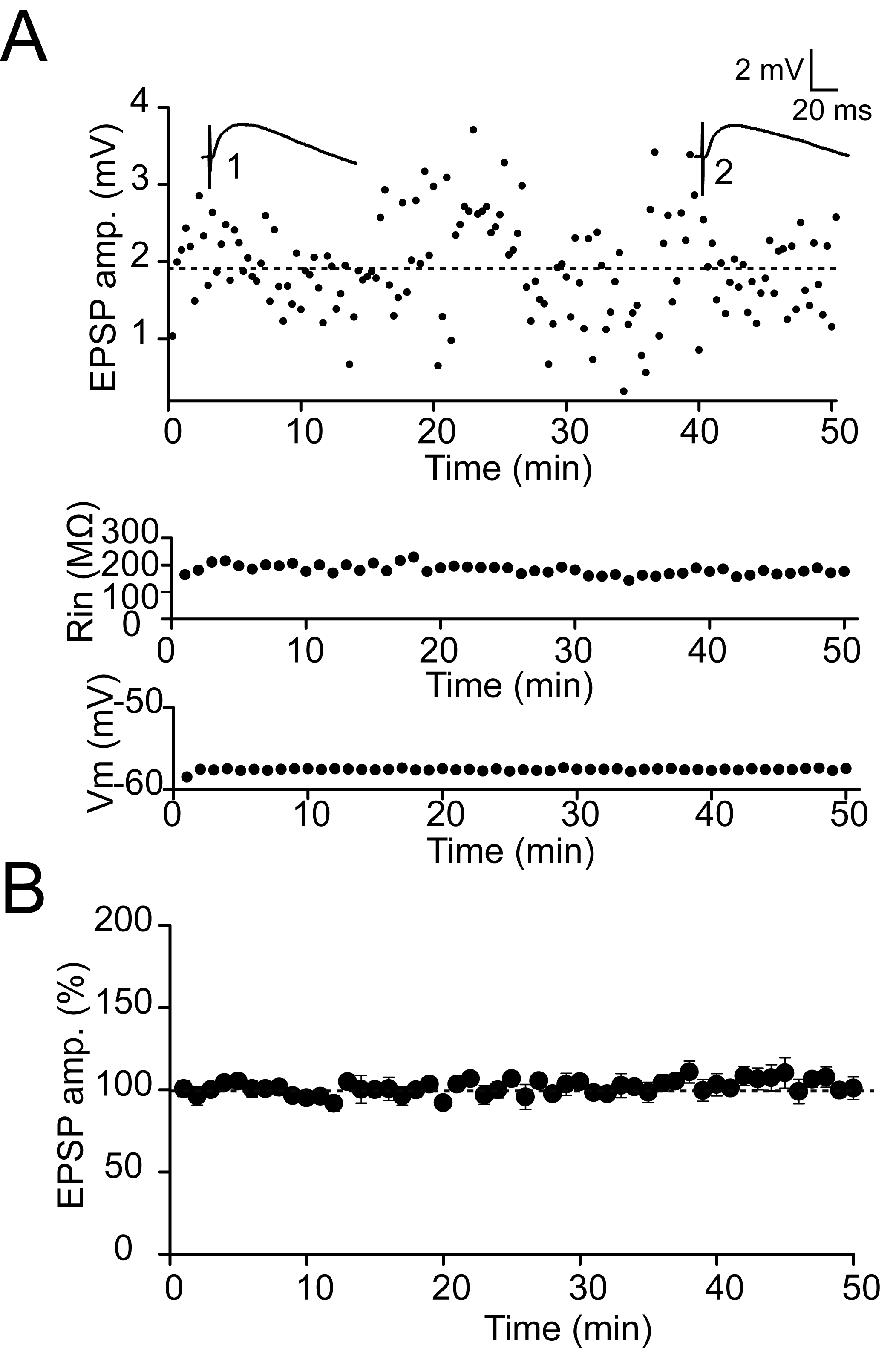

Supplement: Figure S2 — No persistent changes in the EPSPs amplitude were observed when TBS was absent. (A) There was an absence of change in the EPSPs when the TBS was not delivered. Representative traces above the graph show the averaged EPSPs selected at the time-points indicated by the number on the graph. The dashed line indicates the average EPSP amplitude. No obvious changes in the membrane voltage potential (Rin, middle) or input resistance (Vm, bottom) were detected. (B) Summary of the averaged data in experiments as shown in A. The EPSP amplitude was evaluated during the last 10 min and is presented as a percentage of the baseline EPSP amplitude. No rundown of the EPSP amplitude was observed (p>0.05). (TIF) [file pone.0035001.s002.tif]

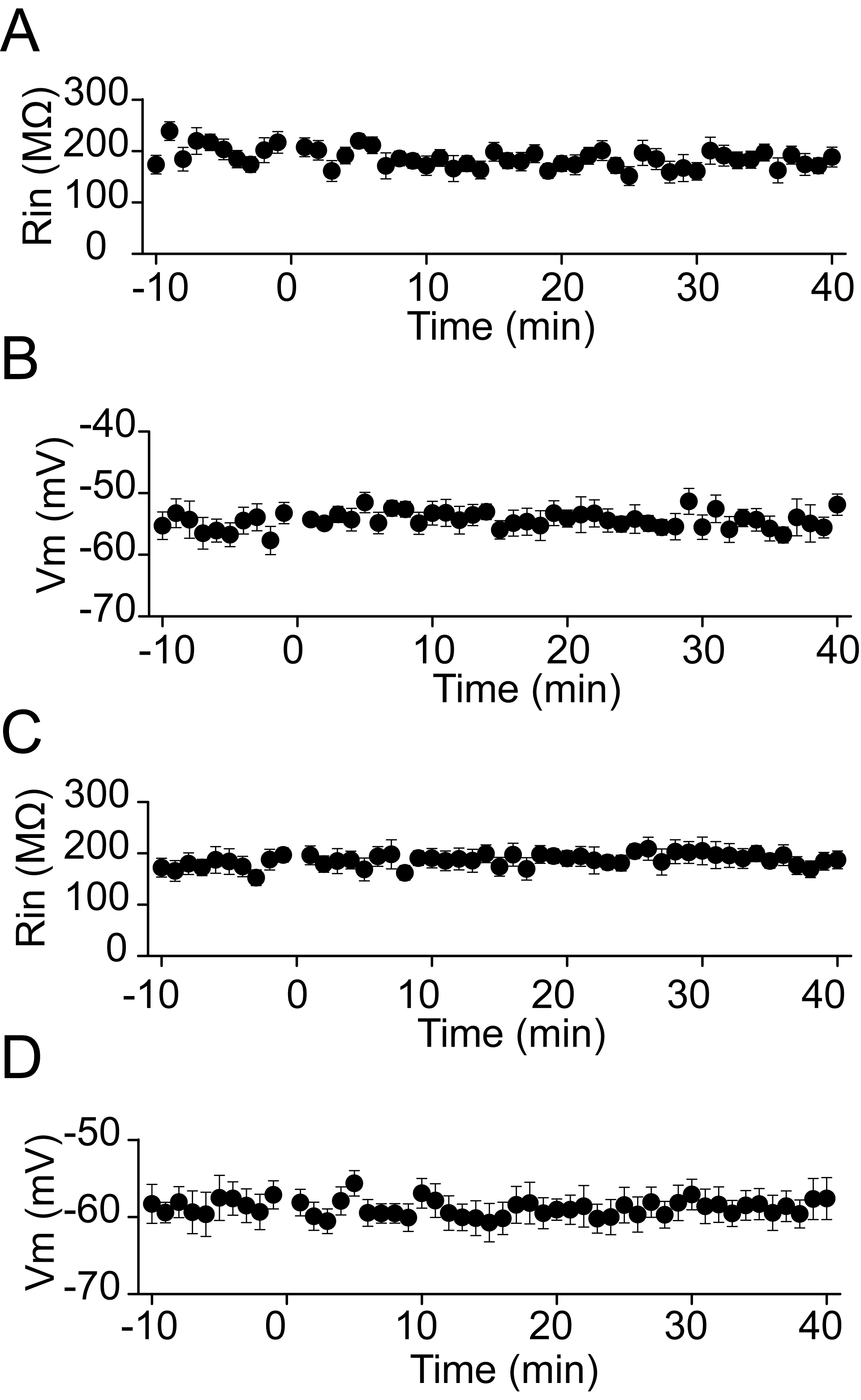

Supplement: Figure S3 — TBS-induced synaptic plasticity was not associated with obvious changes in the input resistance or membrane potential. (A) and (B) The statistical profiles of the changes in the averaged input resistance (A; Rin) and membrane potential (B; Vm) associated with the TBS-induced LTD in GCs. (C) and (D) The statistical profiles of the changes in averaged input resistance (C; percentage of baseline) and membrane potential (D) associated with TBS-induced LTP in GCs. No significant changes in the Rin or Vm were detected during the TBS-induced synaptic plasticity in GCs. (TIF) [file pone.0035001.s003.tif]

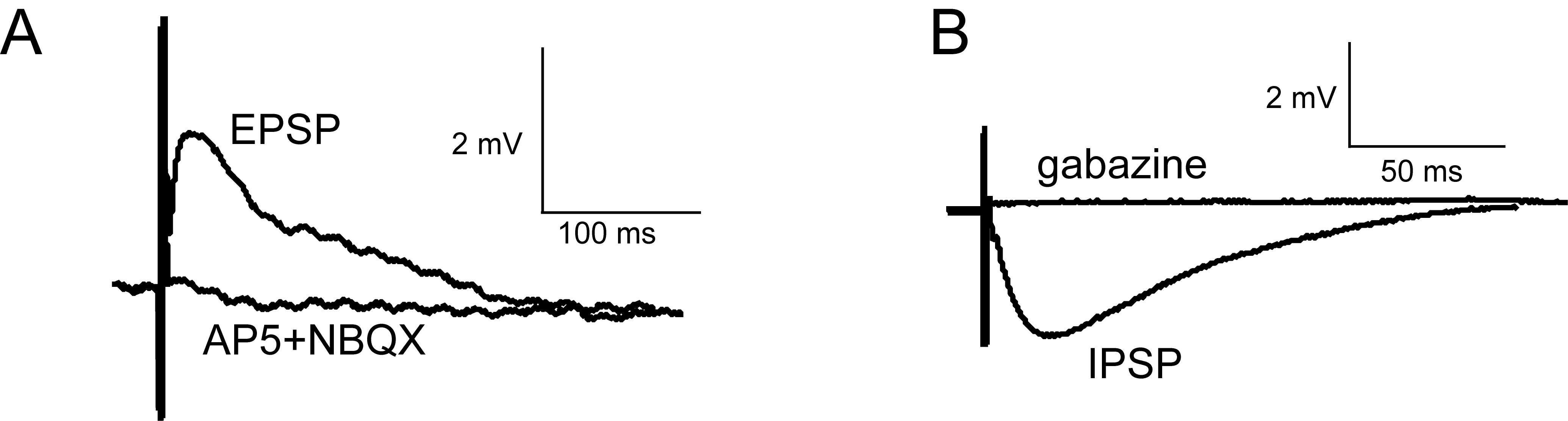

Supplement: Figure S4 — EPSPs and IPSPs recorded in MCs were blocked by an antagonist for glutamate or the GABAA receptor. (A) The EPSPs recorded in MCs were abolished by co-application of NMDA- and AMPA-type glutamate receptors antagonists AP5 (50 µM) and NBQX (20 µM), whereas the IPSPs were abolished by application of the GABAA receptor antagonist GBZ (10 µM), suggesting that they were mediated by the glutamate receptor and GABAA receptor, respectively. (TIF) [file pone.0035001.s004.tif]

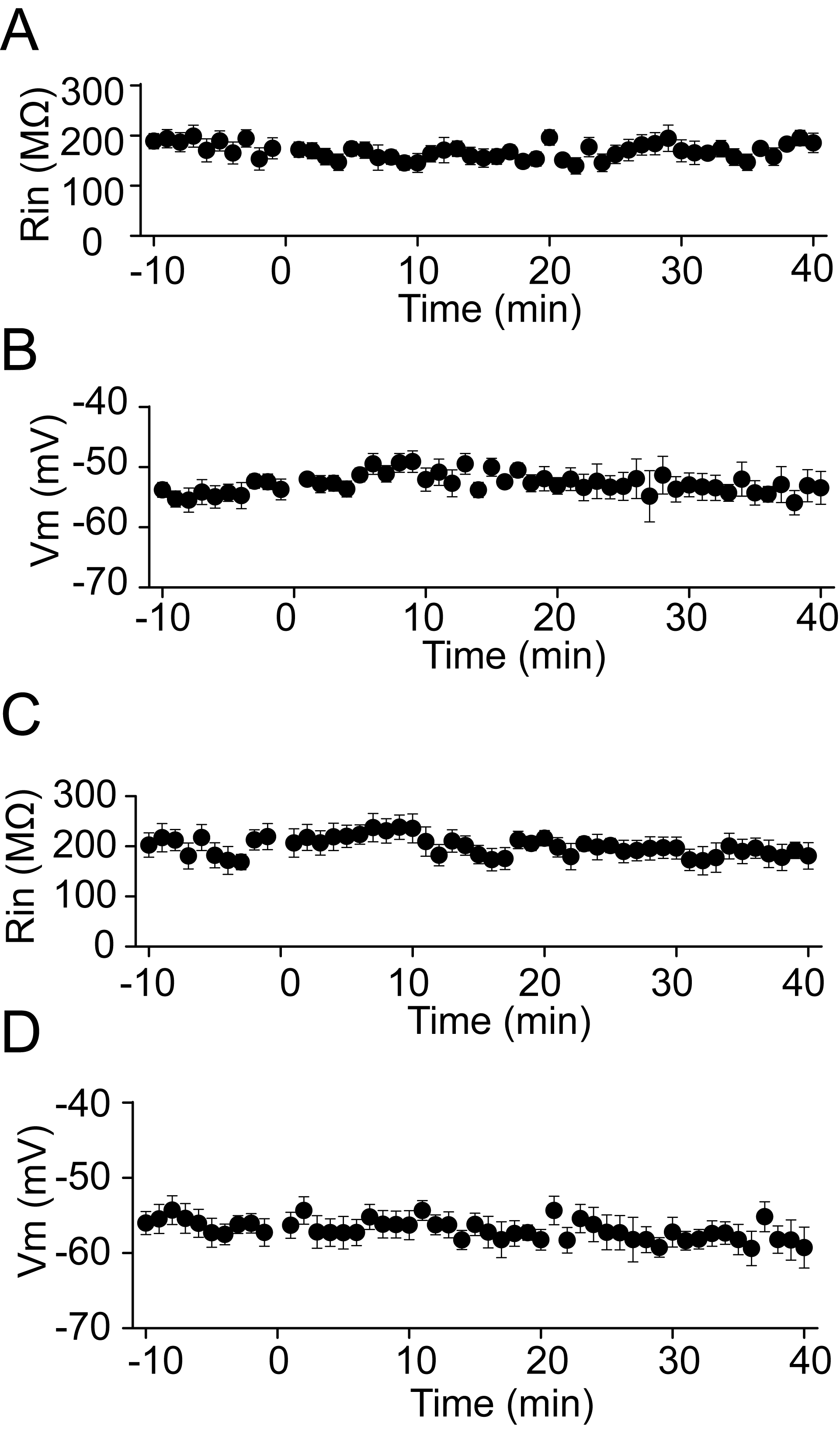

Supplement: Figure S5 — TBS-induced synaptic plasticity was not associated with obvious changes in the input resistance or membrane potential. (A) and (B) The statistical profiles of the changes in the averaged input resistance (A; Rin) and membrane potential (B; Vm) associated with the TBS-induced LTD in MCs. (C) and (D) The statistical profiles of the changes in averaged input resistance (C; percentage of baseline) and membrane potential (D) associated with TBS-induced LTP in MCs. No significant changes in the Rin or Vm were detected during the TBS-induced synaptic plasticity in MCs. (TIF) [file pone.0035001.s005.tif]

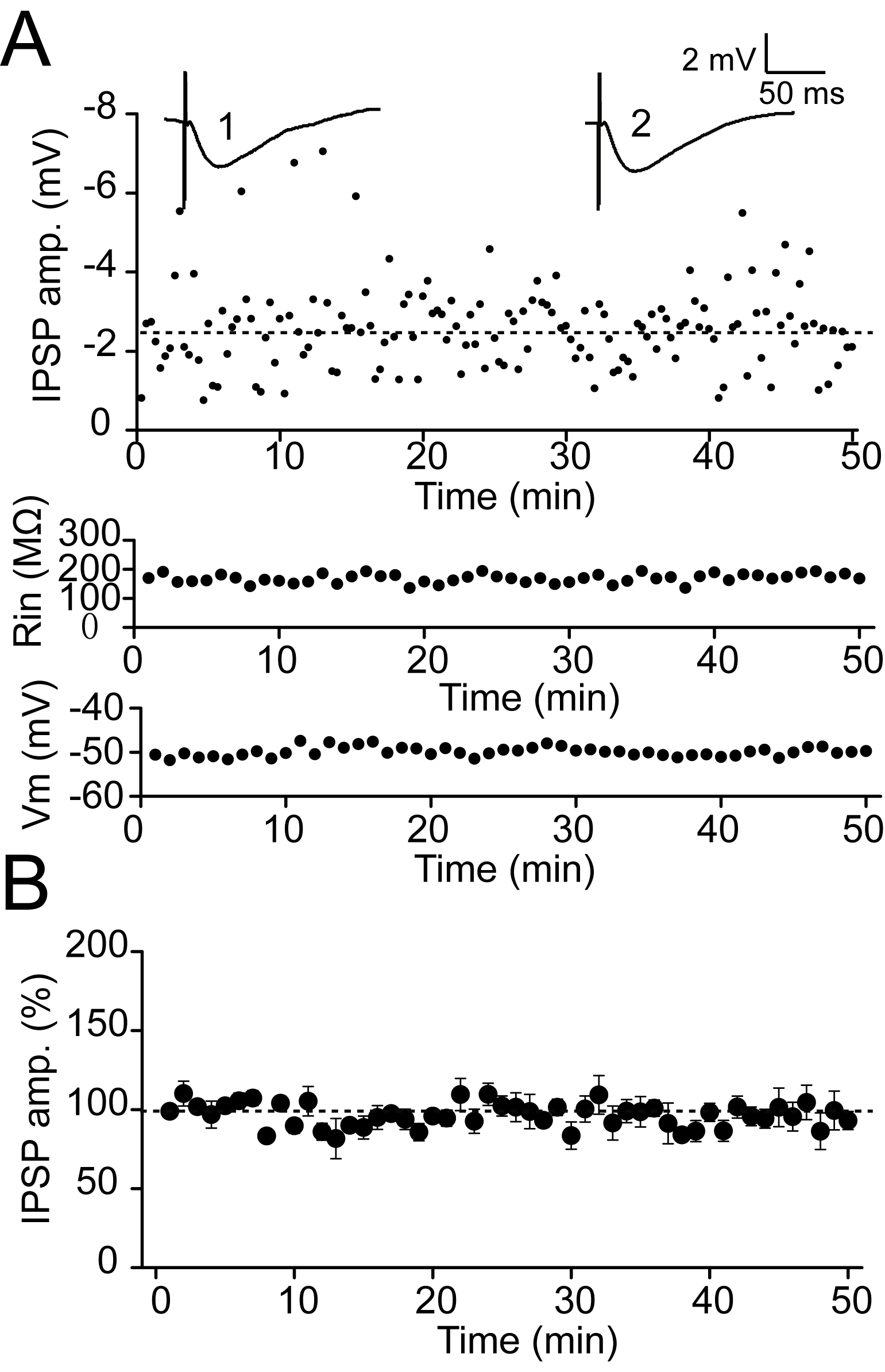

Supplement: Figure S6 — No persistent changes in the IPSPs amplitude were observed when the TBS was absent. Absence of changes in the IPSPs when TBS was not delivered. Representative traces above the graph show averaged IPSPs selected at the time-points indicated by the number on the graph. The dashed line indicates the average IPSP amplitude. No obvious changes in the membrane voltage potential (Rin, middle) or input resistance (Vm, bottom) were detected. (B) Summary of the averaged data in experiments as shown in A. The IPSP amplitude was evaluated during the last 10 min and normalized to the baseline IPSP amplitude. No rundown of the IPSP amplitude was observed. (TIF) [file pone.0035001.s006.tif]

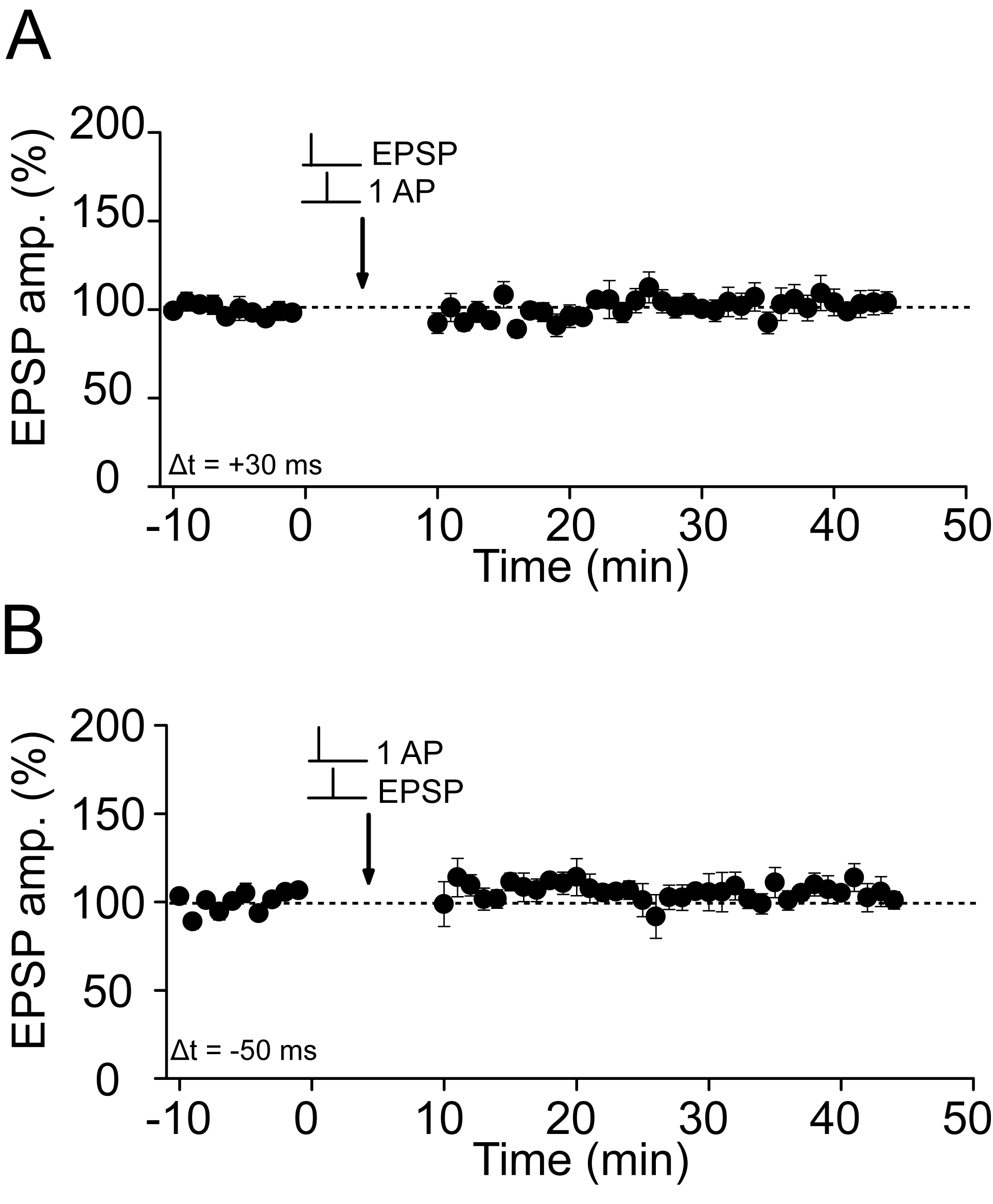

Supplement: Figure S7 — Pairing presynaptic EPSP with single postsynaptic spikes failed to induce any persistent changes in the EPSP in MCs. (A) When repetitive EPSPs preceded the single postsynaptic action potentials induced by injected currents at a +30 ms time window, no long-lasting changes in the EPSPs were detected. (B) When repetitive single postsynaptic action potentials preceded EPSPs at a −50 ms time window, changes in the EPSPs were absent. (TIF) [file pone.0035001.s007.tif]

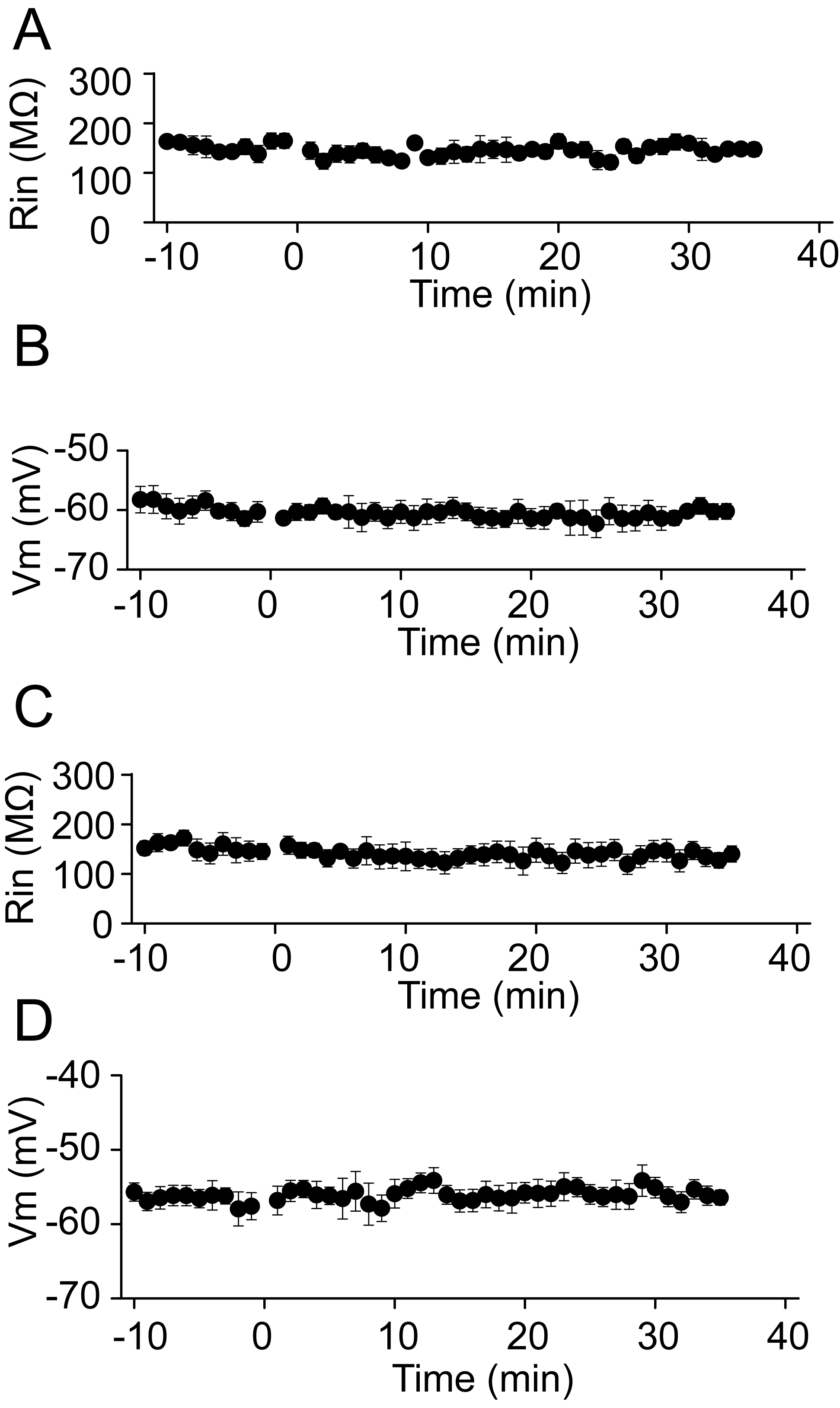

Supplement: Figure S8 — STDP in MCs was not associated with obvious changes in the input resistance or membrane potential. (A) and (B) The statistical profiles of the changes in the averaged input resistance (A; Rin) and membrane potential (B; Vm) associated with the spike timing-dependent LTP in MCs. (C) and (D). The statistical profiles of the changes in the averaged input resistance (C) and membrane potential (D) associated with the spike timing-dependent LTD in MCs. No significant changes in Rin or Vm were detected during the TBS-induced synaptic plasticity in MCs. (TIF) [file pone.0035001.s008.tif]

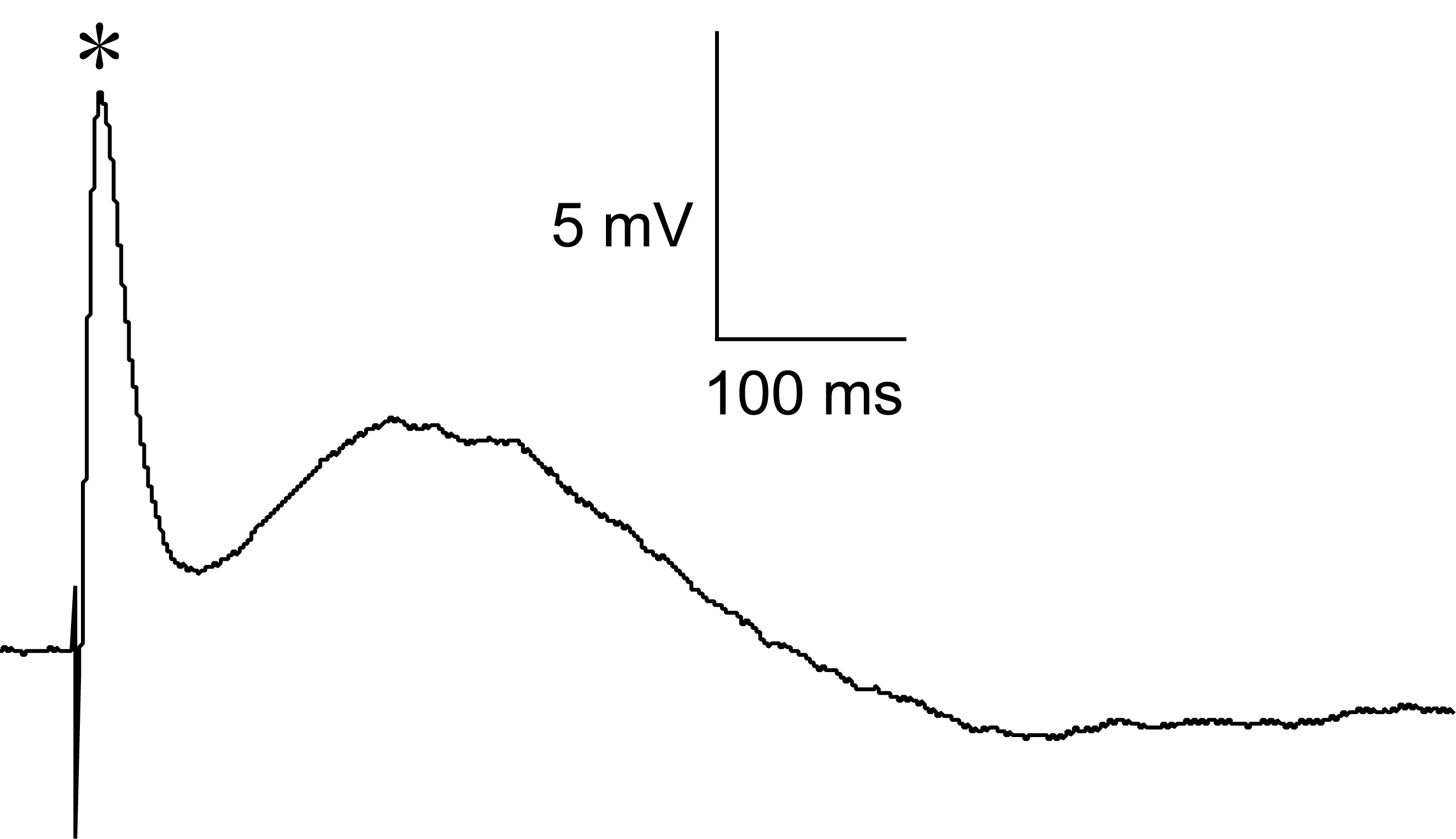

Supplement: Figure S9 — A sample trace showing multiple components in one recording of EPSPs in MCs. The initial peak currents represent monosynaptic responses. (TIF) [file pone.0035001.s009.tif]

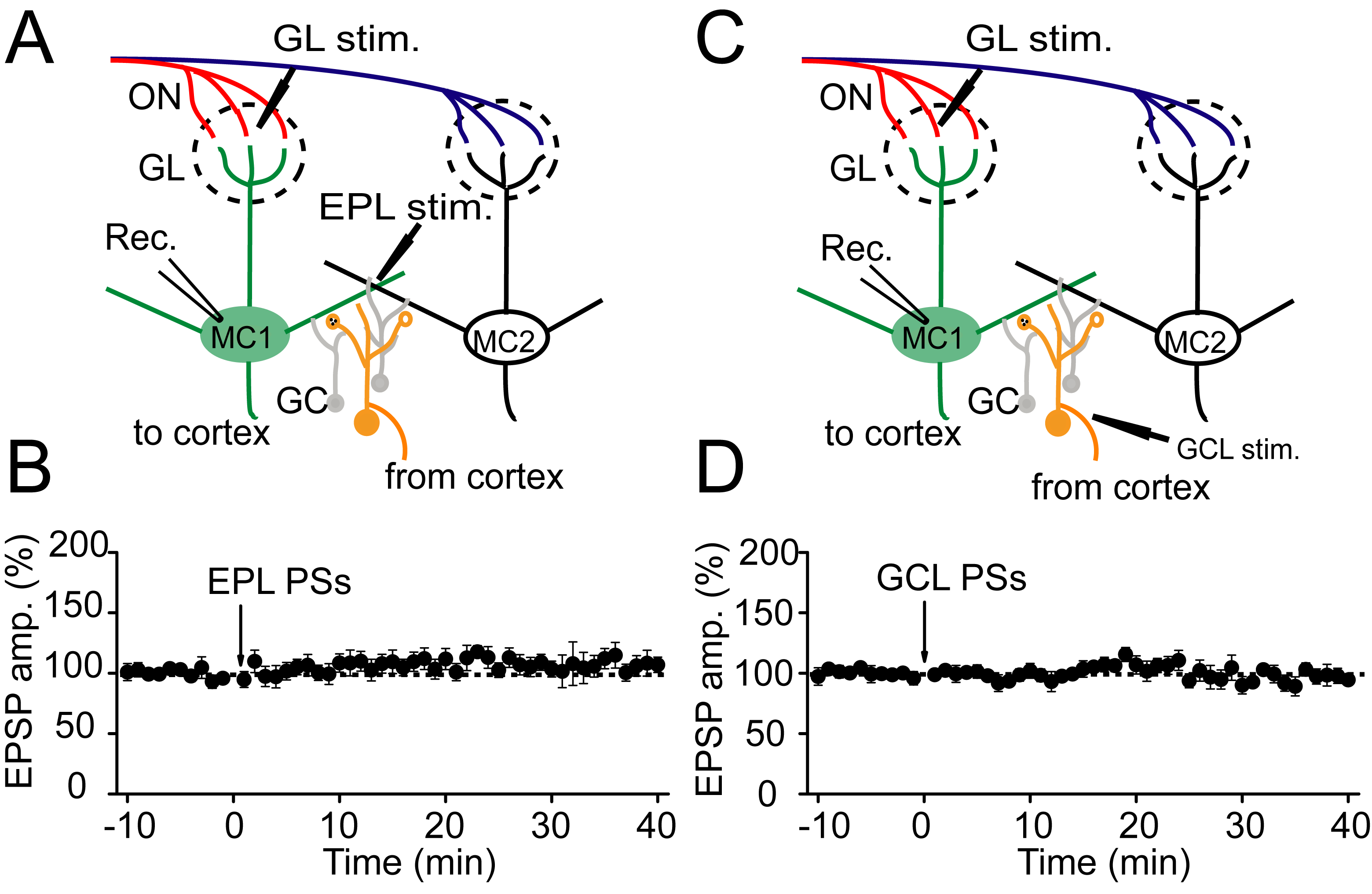

Supplement: Figure S10 — The prior TBS protocol did not affect baseline EPSPs of olfactory inputs. (A) and (C) Schematic of the experimental configuration. The TBS was delivered to distal inputs to the GCs at the EPL (A) or proximal inputs at the GCl (C). (B) and (D) Prior TBS at the EPL (B; EPL PSs; n = 6) or at the GCL (D; GCL PSs; n = 6) did not display an obvious effect on the baseline EPSPs of MCs. (TIF) [file pone.0035001.s010.tif]

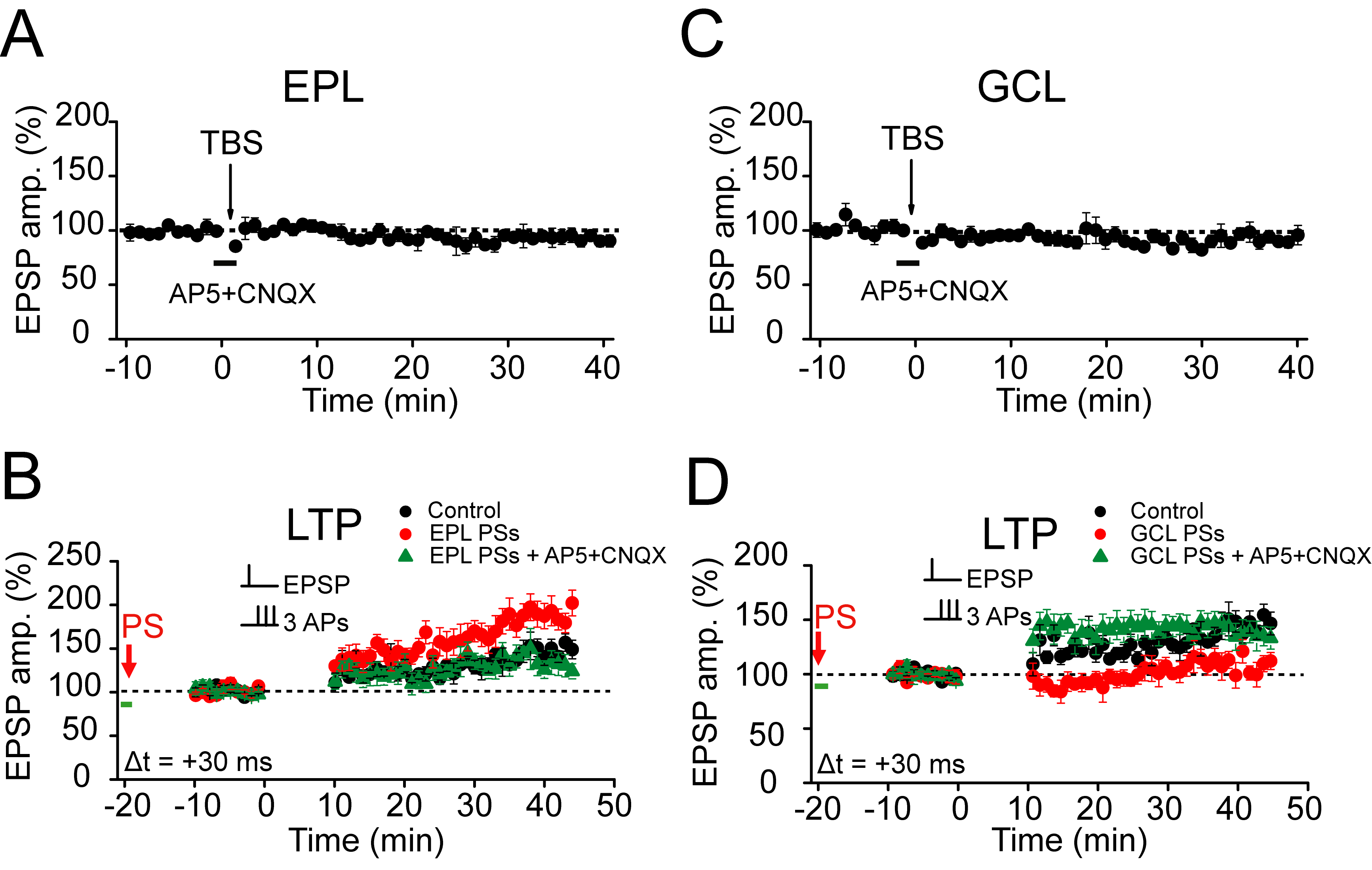

Supplement: Figure S11 — Blocking glutamatergic neurotransmission during TBS abolished plasticity in GCs and reversed the change in STDP in MCs. (A) and (C) Blocking glutamatergic neurotransmission when TBS was delivered onto distal (A; 94.0±2.1%, n = 5; compared with baseline p>0.05) or proximal inputs (C; 91.6±3.5%, n = 4; p>0.05) abolished long-term plasticity in GCs. (B) and (D) Absence of Changes in STDP in MCs when plasticity in GCs was abolished by blocking glutamatergic neurotransmission during TBS onto distal (B; 132.9±8.0%, n = 6; compared with control p>0.05, ANOVA LSD test) or proximal inputs (D; 140.2±5.3%, n = 7; compared with control p>0.05). (TIF) [file pone.0035001.s011.tif]

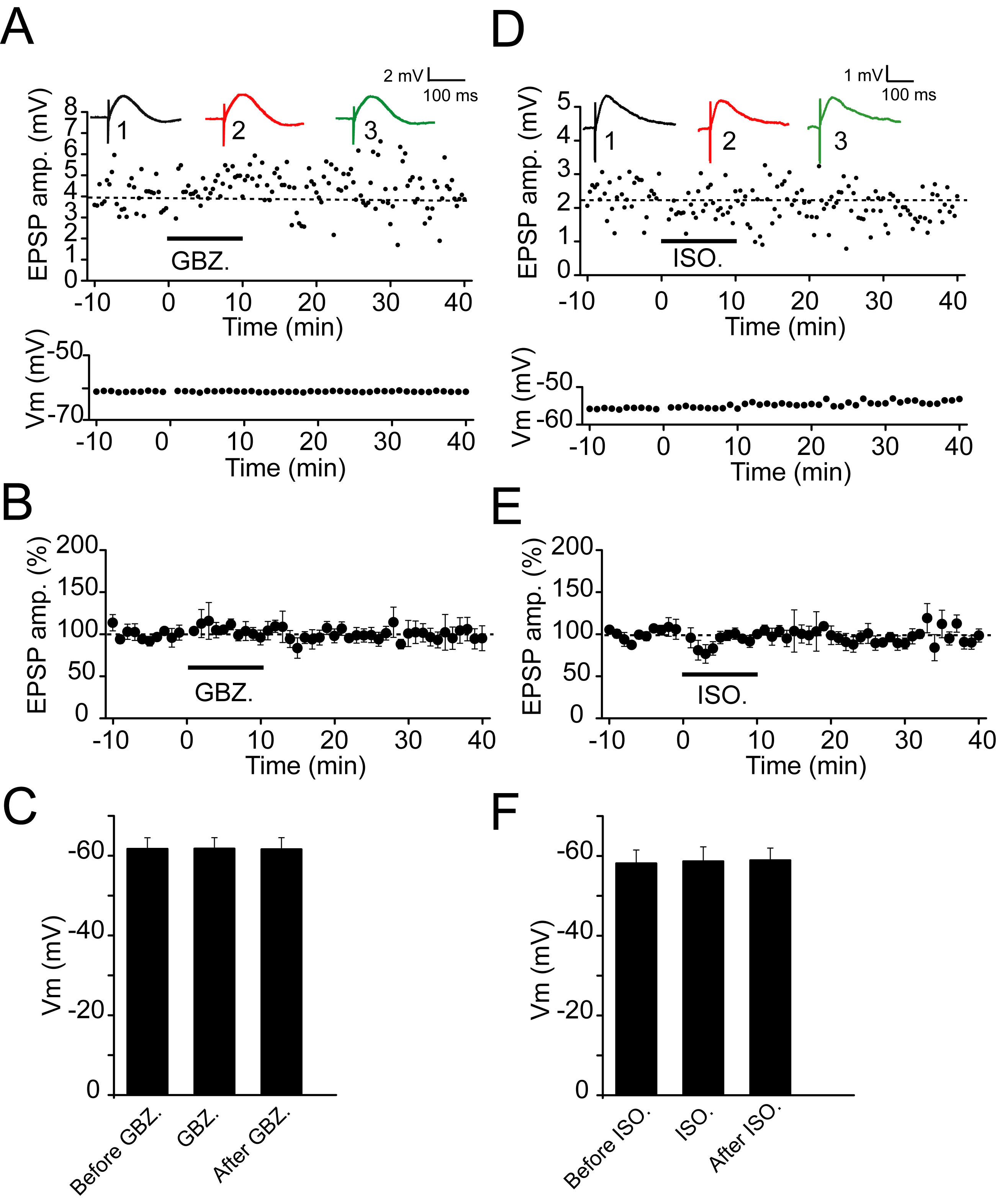

Supplement: Figure S12 — Absence of changes in the synaptic responses and membrane potentials following GBZ or ISO application. (A) GBZ application failed to affect the EPSC amplitude (top) and membrane potentials (Vm; bottom). Examples on the top show the synaptic responses before and after GBZ application. (B) Summary of the data showing the absence of changes in the EPSP amplitude following GBZ (1.5 µM) treatment. (C) Histogram plot showing the absence of changes in the Vm following GBZ treatment (n = 4, p>0.05). (D) The ISO (3.0 µM) did not change the amplitude of the EPSPs. (E) Summary of the data showing the absence of changes in the EPSP amplitude following ISO (3.0 µM) treatment. (F) Histogram plot showing the absence of changes in the Vm following ISO treatment (n = 4, p>0.05). (TIF) [file pone.0035001.s012.tif]

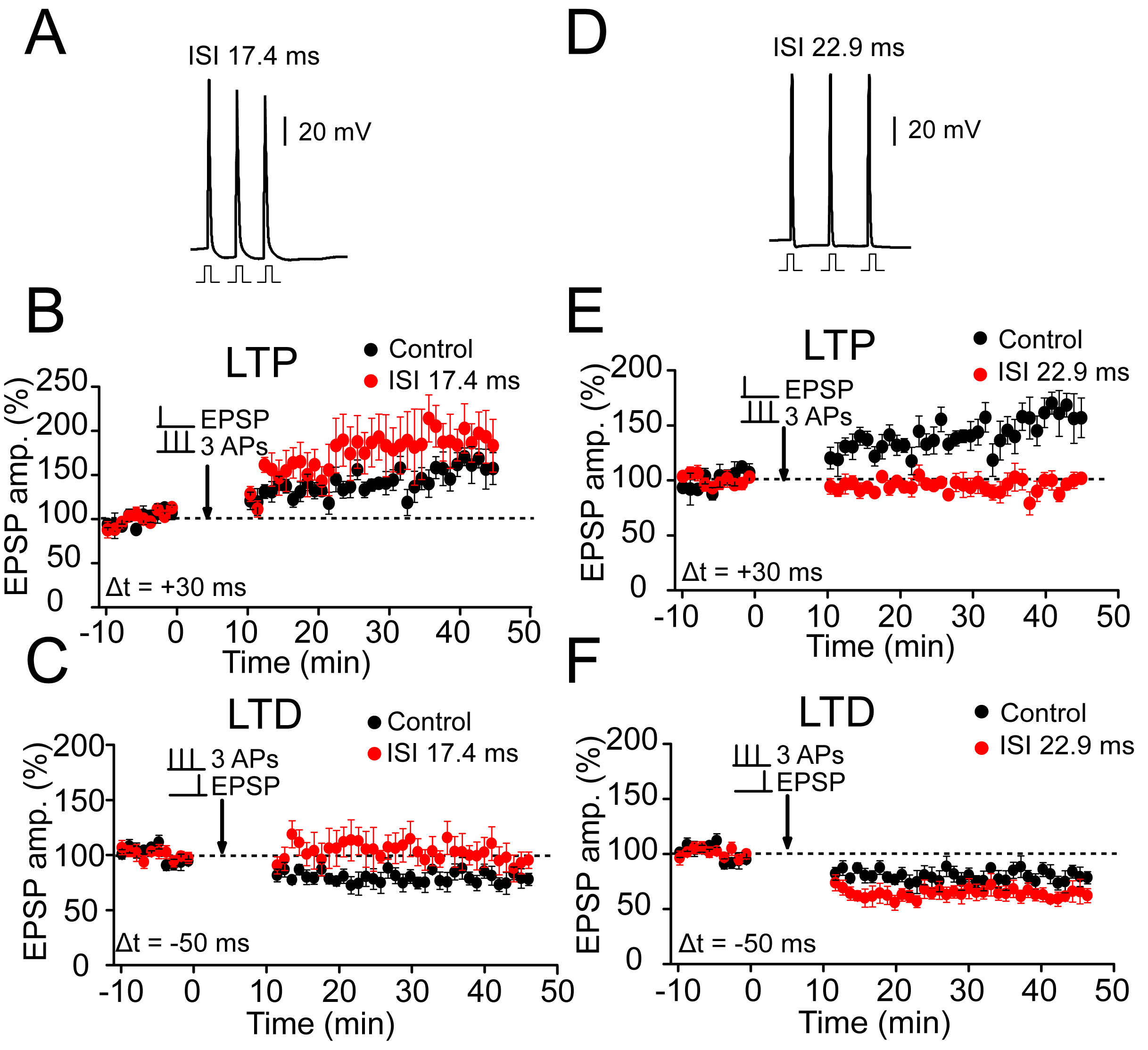

Supplement: Figure S13 — Manipulation of the ISI by adjusting the frequency of spikes mimicked regulation of plasticity by prior TBS. (A) Representative trace of spike bursts evoked by three short current steps with the ISI set at 17.4 ms. (B) Summary of the changes in the EPSP amplitude showing facilitation of the LTP in MCs. When the ISI was set at 17.4 ms by adjusting the frequency of the spikes induced by three short current steps, the repetitive pairing of EPSPs with the bursts at a +30 ms time window (Δt = +30 ms, repeated 60 times) produced a LTP with a greater magnitude (n = 6, p<0.001). (C) Summary of changes in the EPSP amplitude showing a suppression of LTD. When the ISI was set at 17.4, the LTD produced by the pairing protocol at a −50 ms time window (Δt = −50 ms, repeated 60 times) was suppressed (n = 6, p<0.001). (D) Representative trace of spike bursts evoked by three short current steps with the ISI set at 22.9 ms. (E) Summary of the changes in the EPSP amplitude showing suppression of LTP. When the ISI was set at 22.9 ms, the repetitive pairing of EPSPs with the bursts at a +30 time window produced LTP with a decreased magnitude (n = 6, p<0.001). (F) Summary of changes in the EPSP amplitude showing facilitation of LTD. When the ISI was set at 22.9 ms, the LTD with a greater magnitude was produced by the pairing protocol at a −50 ms time window (n = 6, p<0.001). Interestingly, the bidirectional manipulations of the ISI, which mimicked the changes in the ISI produced by the PSs, induced a similar modification of the STDP. (TIF) [file pone.0035001.s013.tif]

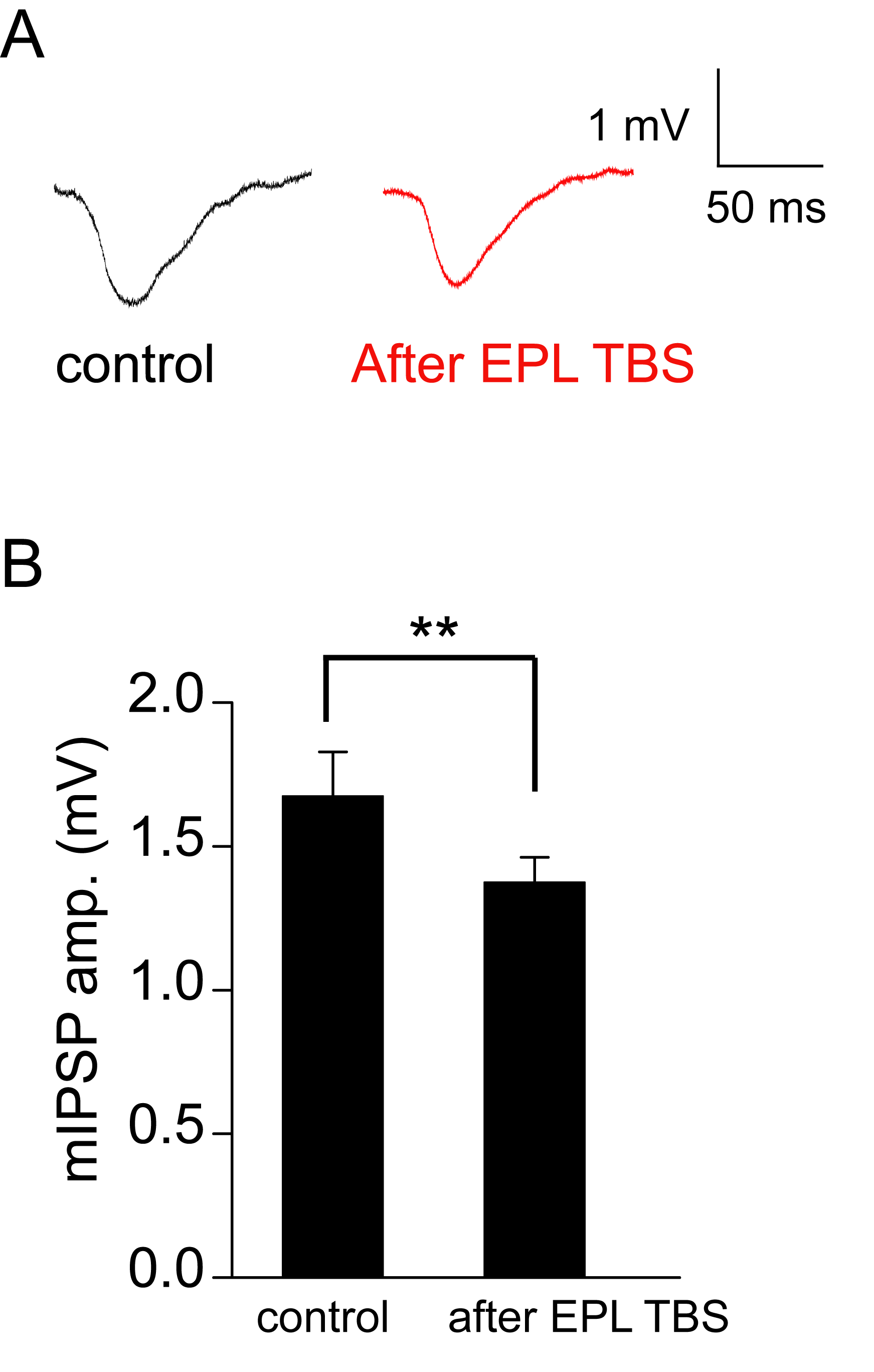

Supplement: Figure S14 — TBS induced tonic inhibition of mIPSPs in MCs. (A) Sample traces before and after TBS displays the change in IPSP amplitude. (B) Summary of the change in the mIPSP amplitude following TBS at the EPL. (TIF) [file pone.0035001.s014.tif]

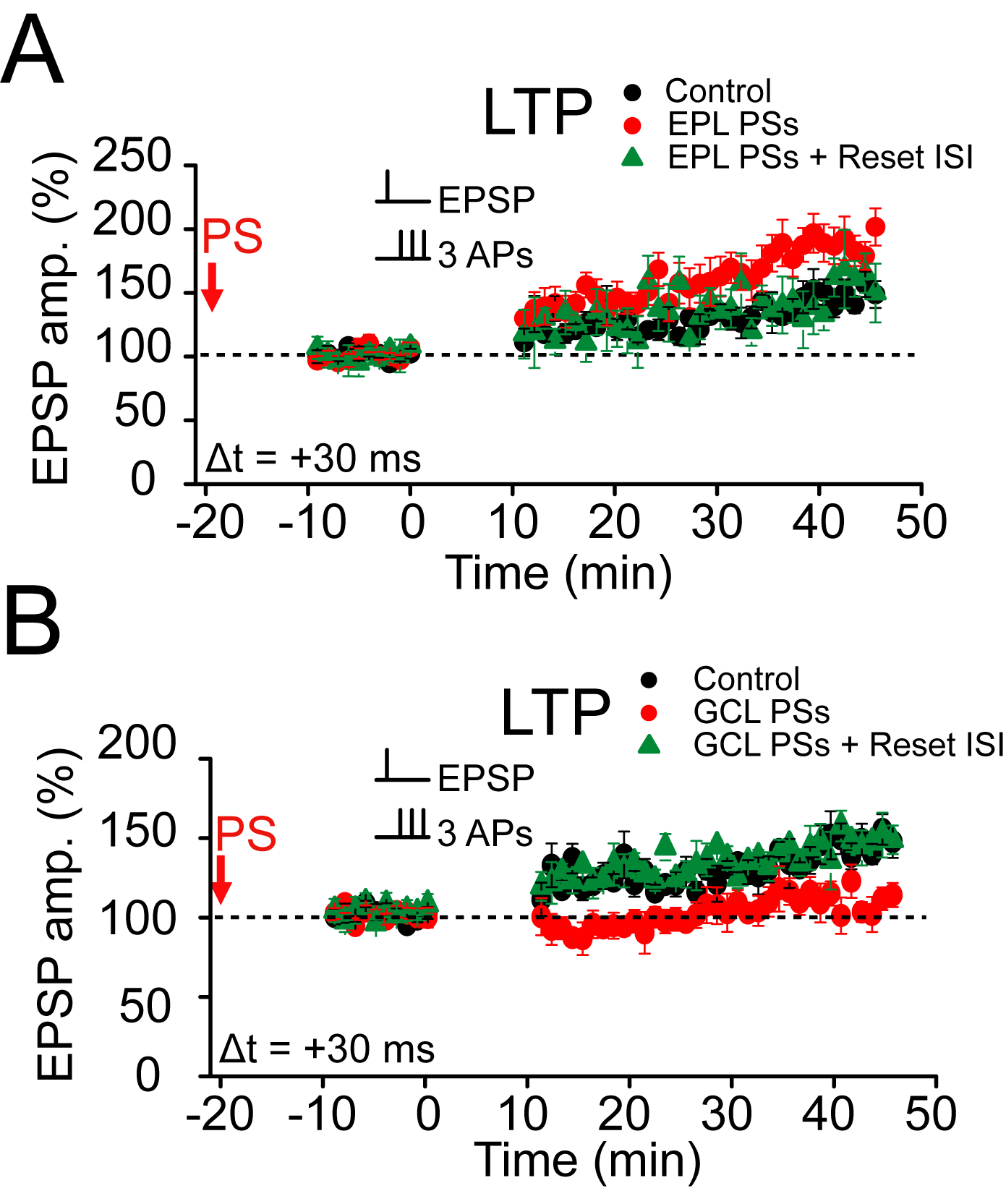

Supplement: Figure S15 — Bidirectional regulation of STDP by resetting the ISI after prior TBS. (A) The EPL priming stimulation (EPL PSs) altered the ISI of the burst induced by a single current injection. This change in the ISI could be reversed by regulating the ISI back to control levels via three individual current injections at a frequency similar to the control. As a result, the LTP was reverted back to control levels. (B) A similar observation was made when both the GCL priming stimulation (GCL PSs) and the resetting of the ISI were performed. The data obtained from the control, EPL PSs and GCL PSs in this figure were taken from Fig. 4B and Fig. 5B for comparison. (TIF) [file pone.0035001.s015.tif]
